# When a model gives you mixed signals: cognitive effects and visual behavior

**DOI:** 10.1007/s44311-025-00022-8

**Published:** 2025-10-06

**Authors:** Amine Abbad-Andaloussi, Marco Franceschetti, Hugo A. López, Clemens Schreiber, Barbara Weber

**Affiliations:** 1 University of St.Gallen, St. Gallen, Switzerland; 2 Technical University of Denmark, Kgs. Lyngby, Denmark; 3 Karlsruhe Institute of Technology, Karlsruhe, Germany

**Keywords:** Ambiguity, Process models, Eye-tracking, Cognitive load, Visual behavior

## Abstract

Ambiguity in business process models can result in multiple interpretations by model readers. This leads to undesirable outcomes such as misunderstandings, unclear allocation of responsibilities, and unexpected behaviors. Despite these potential consequences, the impact of ambiguity on model readers has received limited attention so far. This article presents an eye-tracking study designed to investigate the effects of various types of ambiguity (i.e., layout, semantic, syntactic, and lexical) on readers’ cognitive load, comprehension, and visual associations while interpreting process models. In addition, the study delves into the behaviors of model readers when resolving ambiguity in process models. These behaviors are investigated following a qualitative approach combining both eye-tracking and think-aloud data. The results demonstrate that ambiguities significantly influence cognitive load, comprehension, and visual associations, emphasizing the negative effects of ambiguity. Moreover, the qualitative insights suggest that participants exhibit specific behaviors when trying to resolve ambiguities. These findings underscore the need for advanced mechanisms to detect and mitigate ambiguity in process models.

## Introduction

Business process models are widely used to improve the understanding of business processes by combining information visualization principles with the semantics of formal modeling languages. Several process modeling languages, such as Business Process Model and Notation (BPMN) (OMG 2014) and Petri nets (Reisig 2012), offer formally defined semantics and are commonly adopted for this purpose. However, there is growing recognition that business process models, even with formal semantics, may be prone to ambiguity (Dijkman et al. 2008; Fan et al. 2016; Pittke et al. 2015), raising the need for further investigation into its impact. Ambiguity in a process model is a phenomenon that can lead to multiple valid interpretations of the process: for an example of *lexical* ambiguity affecting activity labels, an activity labeled *Schedule review* can be interpreted as an activity of scheduling a review or as an activity of reviewing a schedule. A recent study in Franceschetti et al. (2023) examined ambiguity across different artifacts within the Business Process Management (BPM) lifecycle, including informal specifications, models, and event logs. The study highlighted the pervasive nature of ambiguity in process models through examples drawn from literature and public datasets. As process models are frequently adopted as a means of communication between stakeholders, the existence of multiple valid interpretations can undermine their communication effectiveness due to the potential mismatch between a reader’s interpretation and the modeler’s intended message. Indeed, as detailed in Franceschetti et al. (2023), ambiguity in process models entails the risk of misunderstandings, unclear responsibilities, unexpected behaviors, as well as cascading effects across other process-related artifacts managed in the BPM lifecycle. Consequently, ambiguity in process models can compromise recognized benefits of adopting Process-aware Information Systems such as clear process-based communication between stakeholders, increased efficiency, reduced redundancies, and monitoring support (Dumas et al. 2018), by diminishing these benefits (Kindler 2009; Oppl 2016). Therefore, correctly comprehending process models is imperative for the successful engineering and operation of a Process-aware Information System (Oppl 2016).

Previous research has examined various types of ambiguities and strategies to reduce them in business process models at design time (cf. Fan et al. (2016); Mendling et al. (2010b); Pittke et al. (2015)). However, to the best of our knowledge, no prior study has validated the impact of ambiguity on the cognitive and behavioral aspects of process model readers. On the one hand, prior studies have explored the impact on these aspects in relation to model complexity (Petrusel et al. 2017), model quality (Heggset et al. 2015), notational deficiencies in modeling languages (Figl et al. 2013), or reader-specific attributes like modeling experience and process knowledge (Mendling et al. 2012). On the other hand, the specific impact of ambiguity on the cognitive and behavioral aspects remains, to date, unexamined. Unlike aspects such as model correctness, ambiguity is characterized by the potential for multiple equally valid interpretations. While in certain contexts, such as normative processes, this feature is intentionally incorporated to provide flexibility in model interpretation, in other contexts ambiguity can have an unintended negative impact as it generates confusion (Franceschetti et al. 2023). This study focuses on understanding how this unique characteristic, which can lead to multiple interpretations, influences the cognitive and behavioral aspects of process model readers.

Particularly, in this study, we address the challenge of *measuring the cognitive impact* of ambiguity in process models on readers’ cognitive load (i.e., mental effort), comprehension, and visual associations (i.e., shifts of attention between model elements, which suggests an increased mental demand for integrating information, cf. Bera et al. (2019)). Additionally, we *explore the visual behavior* of model readers when confronted with ambiguity. To achieve this, we use eye-tracking, a method that has proven effective in previous research on process model comprehension tasks, offering valuable insights into the cognitive and behavioral aspects of model readers (cf. Bera et al. (2019); Petrusel and Mendling (2013); Petrusel et al. (2016); Wang et al. (2022); Winter et al. (2023)).

Overall, we address the following research questions:

- RQ1. How do different ambiguities affect model readers’ cognitive load, comprehension and visual associations?
- RQ2. What patterns of visual behavior do model readers exhibit when resolving ambiguities in process models?

This paper extends our prior work presented in Franceschetti et al. (2024), which investigated RQ1, by additionally investigating RQ2. To answer RQ1, we investigated the eye movements of model readers while they performed different comprehension tasks on process models with and without ambiguities. To answer RQ2, we performed a qualitative study in which we observed the eye movements of model readers when confronted with ambiguity, and triangulated these observations with the verbal information provided by the model readers during retrospective think-aloud sessions. Our results demonstrate the usefulness of eye-tracking to detect ambiguity and that ambiguous process models lead to higher cognitive load, challenged comprehension, and increased visual associations (RQ1) when compared to process models without ambiguities. Moreover, our qualitative study revealed five distinct visual behavior patterns that emerge when model readers are confronted with ambiguity, each associated with varying levels of visual attention, ranging from random to highly focused (RQ2). Having demonstrated the negative effects of ambiguity and how it influences model readers’ cognitive aspects and visual behavior, future work could focus on developing methods and techniques to support model readers by automatically detecting ambiguities in process models, or assisting them in focusing their attention on model elements that provide disambiguation cues. Our results also stimulate further studies on the impact of ambiguity in relation to expertise and on disambiguation strategies.

This paper is structured as follows: in Background and related work section, we recall background concepts and formally define ambiguities in process models. In Research method section, we report on our study design. In Findings section, we report on the findings. In Discussion section, we elaborate on a discussion of the findings, the implications of the study results, and threats to validity. In Conclusion section, we conclude the paper.

## Background and related work

In this section, we first establish the theoretical foundations of ambiguity in BPM, formalizing four ambiguity types found in process models (cf. Ambiguity in process models section). We then set the theoretical foundations on cognitive theories relevant to our empirical study, setting the underpinnings for and motivating our study design (cf. Cognitive load, comprehension, and visual associations section). Finally, we provide an overview of qualitative approaches investigating modelers’ visual behavior when engaging with process models (cf. Qualitative analysis of visual behavior during process comprehension section).

### Ambiguity in process models

In general, ambiguity in BPM refers to the potential for a business process representation to yield multiple admissible interpretations. According to the characterization provided in Franceschetti et al. (2023), specifically ambiguity *in process models* may arise from intrinsic factors (i.e., specific to the modeling language, such as the inability to represent the resources perspective in standard Petri nets) or extrinsic factors (i.e., related to the modeling task, such as an underspecified gateway condition by an inexperienced modeler not validated by the modeling tool).

As process models are formalized using modeling languages that combine both textual and graphical elements, they encompass the following aspects: syntactic aspects in relation to the use of the modeling language grammar, semantic aspects in relation to the modeled behavior, lexical aspects in relation to textual elements, and layout aspects in relation to the graphical presentation. Since all these aspects influence a model reader’s interpretation of the model, in this study we aim to extend the aforementioned ambiguity characterization (Franceschetti et al. 2023) by defining layout^1^, semantic, syntactic, and lexical ambiguities in process models. To the best of our knowledge, these types of ambiguities are still not clearly defined in the BPM literature. To define these ambiguities in the BPM context, we considered two main options.

On the one hand, we could adopt corresponding definitions from the field of linguistics (Berry And Kamsties 2004; Fortuny And Payrató 2024; Sennet 2011). However, such definitions apply specifically to natural language, which is typically presented in oral or textual form, and do not fully apply to the diagrammatic nature of process models, which combine both textual and graphical elements. Thus, definitions from linguistics do not fully capture the ways in which ambiguity arises in business process models, since process model interpretation depends not only on natural language but also on diagrammatic conventions, domain mappings, and layout (Figl 2017; Haisjackl et al. 2015). Moreover, the distinction between the different types of ambiguity in linguistics does not fully align with the one from our BPM field. For example, the BPM literature includes layout characteristics as part of pragmatic aspects (Haisjackl et al. 2015), whereas in linguistics pragmatics concerns contextual and inferential aspects of communication related to speaker intent (Fortuny And Payrató 2024).

On the other hand, we could also rely on prior BPM literature that has dealt with some of these ambiguities (cf. Amna and Poels (2022); Fan et al. (2016); Haisjackl et al. (2015); Leopold et al. (2010); Mendling et al. (2010b); Pittke et al. (2015)), but has not achieved consolidated BPM-specific definitions. Moreover, prior literature that has studied model quality aspects in BPM could also be relevant (cf. Krogstie (2012, 2016); Lindland et al. (1994)) as the violation of quality guidelines often leads to ambiguity. However, ambiguity and quality are different concepts. Indeed, while ambiguities are often associated with quality issues, in general ambiguity is not reducible to quality: even a formally correct and high-quality model may give rise to ambiguity. This is observed, for example, in models of legal processes, which are of high-quality but can *intentionally* be designed ambiguous to allow for a flexible application of the law (Hildebrandt 2018; Slaats et al. 2013). As a result, we decided to develop our own BPM-specific definitions of ambiguity, drawing from the non-consolidated definitions in existing BPM literature, while also being informed by process model quality frameworks.

#### Definition 1

(Layout Ambiguity) (Adapted from Amna and Poels (2022); Haisjackl et al. (2015)) Layout ambiguity is a phenomenon that occurs at the layout level causing a process model to lack clarity in one or more process perspectives, allowing for multiple interpretations without affecting the behavior of the executable process model.

**Fig. 1:**
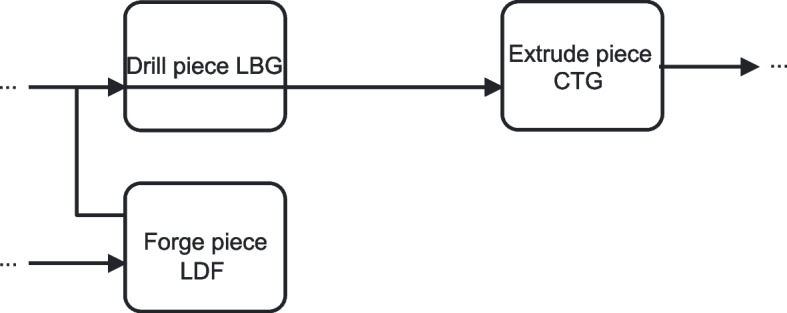
BPMN fragment of a process with layout ambiguity

In our BPM-specific view, layout ambiguity refers to ambiguity that arises not from the structure or semantics of a process model per se, but from aspects related to the presentation and layout of the model. This includes, for example, overlapping edges, poorly aligned flows, or ambiguous visual groupings–which may allow for multiple plausible interpretations of the process elements. Figure 1 illustrates an example of layout ambiguity. In the example, overlapping control flow edges between the activities can be observed. This overlap allows for multiple interpretations of the precedence constraints between the activities. In the specific model fragment, it is unclear whether activity *“Forge piece LDF”* is followed by *“Drill piece LBG”* or by *“Extrude piece CTG”*. The potential of ambiguity arising from the presentation layout was discussed in Petre (1995), however without touching upon the impact on a reader’s cognitive and behavioral aspects. Process model comprehension in relation to layout aspects was further studied in subsequent works such as Figl (2017); Haisjackl et al. (2015); Petrusel et al. (2016). Still, the effect on comprehension specifically arising from ambiguity in the layout, i.e., the multiple possible interpretations yielded by the model layout, was not considered by these studies. Therefore, the question about how (layout) ambiguities affect model readers’ cognition remained, so far, unaddressed.

#### Definition 2

(Semantic Ambiguity) (Adapted from Fan et al. (2016); Haisjackl et al. (2015); Krogstie (2012)) Semantic ambiguity is a linguistic phenomenon related to the usage of a modeling language that occurs as a consequence of a process model lacking validity (i.e., all statements in the model are correct and related to the process) or completeness (i.e., there is a one-to-one mapping between model constructs and domain concepts), or of differences in domain and context knowledge between model creators and readers. It allows for multiple interpretations by model readers due to the readers not being able to establish clear mappings between model constructs and domain concepts.

**Fig. 2:**
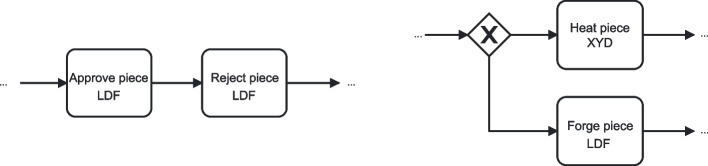
BPMN fragments of processes with semantic (left) and syntactic (right) ambiguities

In our BPM-specific view, semantic ambiguity refers to situations where elements of a process model can be interpreted in more than one way with respect to the (formal) process model behavior, in relation to the underlying business domain. This ambiguity may result from deficiencies in semantic quality such as lack of validity or completeness, but can also arise in formally correct models due to differences in domain knowledge or context among readers. Unlike semantic ambiguity in linguistics, which focuses on multiple meanings of words or phrases, semantic ambiguity in BPM encompasses the broader challenge of aligning model elements with a shared domain understanding. Our focus lies on the interpretive consequence: that different readers may assign different meanings to the same model fragment, regardless of its formal correctness. An example of semantic ambiguity in relation to validity is illustrated in Fig. 2 (left). Here, the sequence *“Approve piece LDF”* – *“Reject piece LDF”* is presented. Simply applying common sense, a model reader would naturally assume that a piece is either approved or rejected, since these verbs express opposite actions. Therefore, in the example it is unclear whether the two activities refer to different pieces or the activities are not supposed to be both executed in the same trace. An example of semantic ambiguity in relation to completeness borrowed from Fan et al. (2016) is that of a single activity *“Send request”* used to model two distinct types of requests in an online auction–from the seller to initiate the auction and a buyer to join the auction. The lack of a one-to-one mapping between domain concepts (two distinct activities) and model elements (one activity) results in a loss of the activity semantics which makes it unclear which request the modeled activity refers to. Semantic ambiguity in process models was studied in Dijkman et al. (2008), specifically focusing on BPMN models. Semantic issues deriving from process model quality issues were investigated in Haisjackl et al. (2015); Krogstie (2012). To reduce semantic ambiguity in process models, a possible approach is to leverage semantic information about the process domain represented through ontologies, as proposed by the authors in Fan et al. (2016). In the examples, the approach helps to (i) validate the activity arrangement, detecting that *“Approve piece LDF”* and *“Reject piece LDF”* in the sequence correspond to mutually exclusive ontology classes, and (ii) identify *construct excess*, detecting the mismatch between one modeled activity and two separate ontology classes (cf. Fan et al. (2016)).

#### Definition 3

(Syntactic Ambiguity) (Adapted from Amna and Poels (2022)) Syntactic ambiguity is a grammatical phenomenon that occurs when a fragment of a process model *M* formalized in a modeling language $\mathcal {L}$ can be parsed using more than one grammatical structure of $\mathcal {L}$, allowing for multiple possible interpretations of *M*.

In our BPM-specific view, syntactic ambiguity is concerned with the parsing of the modeling language constructs. Figure 2 (right) illustrates an example of syntactic ambiguity. In the figure, the control flow splits at an XOR-gateway, which has no conditions attached. Therefore, the gateway can be interpreted either as an underspecified (i.e., with unknown condition) XOR-gateway, which makes the subsequent activities mutually exclusive, or as an incorrectly assigned AND-gateway, which enforces that the subsequent activities are both executed in all traces. Syntactic ambiguity in process descriptions was investigated in Amna and Poels (2022), while the potential emergence of ambiguity due to syntactic quality deficiencies in BPMN models was investigated in Haisjackl et al. (2015). The relation between syntactic aspect of process models and model understandability was discussed in Corradini et al. (2018), along with the proposal of a set of modeling guidelines to avoid the emergence of syntactic ambiguity. Despite these studies, to the best of our knowledge, the relation between syntactic ambiguity and the cognitive and behavioral aspects of model readers remains, to date, unaddressed.

#### Definition 4

(Lexical Ambiguity) (Adapted from Pittke et al. (2015)) Lexical ambiguity is a linguistic phenomenon related to the usage of the natural language that occurs when a textual label in a process model can be interpreted in multiple ways.

According to the studies presented in Corradini et al. (2018); Pustejovsky (1998), lexical ambiguity in BPM can be caused by the use of abbreviations, homonyms, synonyms, and polysemic words (i.e, words with multiple meanings). For example, consider an activity labeled *“Receive report”* followed by an activity labeled *“Evaluate summary”*. Here, it is unclear whether the terms “report” and “summary” are used as synonyms and refer to the same data object, or they refer to different data objects. The use of specific labeling styles, such as passive voice or noun form of verbs, might as well lead to lexical ambiguity (Mendling et al. 2010b). Consider the example borrowed from Pittke et al. (2015) of an activity labeled *“Plan integration”*: the activity could be interpreted either as the planning of some integration, or as the integration of some plan. Prior work from Mendling et al. investigated the usage of activity labels and the resulting lexical ambiguity (Mendling et al. 2010a, b). These studies resulted in guidelines (Mendling et al. 2010c), refactoring recommendations (Leopold et al. 2010), and automatic approaches to detect and resolve lexical ambiguity in process models (Pittke et al. 2015). To the best of our knowledge, however, these studies did not explicitly investigate the impact on the specific cognitive and behavioral aspects that we set to measure in this paper.

### Cognitive load, comprehension, and visual associations

In this section, we provide background and set the theoretical underpinnings to investigate the impact of different ambiguities in process models on readers’ *cognitive load*, *comprehension*, and *visual associations*. This in turn will allow us to address RQ1 (cf. Introduction section).

*Cognitive Load.* Cognitive load denotes the workload imposed on the human working memory during tasks requiring mental processing (Sweller 2011). The Cognitive Load Theory (CLT) discerns three types of cognitive load: Intrinsic, Extraneous, and Germane (Sweller 2011; Chen et al. 2016). *Intrinsic load* emerges from the essential complexity of the process model, which is inherent to the encoded process specifications. *Extraneous* load, in turn, emerges from the accidental complexity of the process model, which is typically associated with the way the model is presented to the user. Finally, *Germane* load arises from the difficulty to integrate the information extracted from the model with ones’ mental schema in order to develop an overarching understanding of the process.

Typically, readers attempt to identify, in the material at hand, features that signal association with their pre-existing mental schemas (An 2013). This identification facilitates schema activation, defined as the process through which individuals retrieve and apply previously acquired knowledge structures from memory to interpret new information (An 2013). For instance, when reading a process model, they may associate an XOR gateway with their pre-existing schema related to mutual exclusion. However, when ambiguities are present, readers may struggle to identify and activate the appropriate schema because multiple interpretations become plausible. As depicted in Fig. 2 (right), an XOR gateway with unlabeled outgoing edges creates ambiguity. Readers might either rely on contextual information to select the most suitable branch, hence associating the underspecified XOR gateway with their mutual-exclusion schema. Alternatively, they can interpret the XOR gateway as an AND gateway, where unlabeled outgoing edges make sense, hence associating the gateway with their pre-existing schema on concurrency. This suggests that ambiguities can challenge the integration of new information with existing schemas, consequently increasing germane cognitive load. This proposition is supported by Campbell’s work (Campbell 1988). In his literature review, Campbell identified several characteristics of complex tasks that impose high mental demands (i.e., cognitive load) on readers. One such characteristic is the presence of uncertain or conflicting information within an artifact. Another is the availability of multiple viable solutions to the task. This, in turn, increases readers’ cognitive load as they must look for additional information, assess alternatives, and make decisions among similarly viable options.

Existing literature proposed several measures aimed at estimating readers’ cognitive load (Figl 2017; Figl et al. 2024; Figl And Laue 2015; Abbad-Andaloussi et al. 2023a; Wang et al. 2022; Schreiber et al. 2024; Zugal 2013; Weber et al. 2021). Among them, self-reported measures rely on individuals’ own assessment of perceived difficulty (Figl 2017; Figl And Laue 2015; Abbad-Andaloussi et al. 2023a; Wang et al. 2022; Schreiber et al. 2024; Zugal 2013), which for example can be rated by readers using a 5-point Likert scale (ranging from 0: “very easy” to 4: “very difficult”) after completing a task (Schreiber et al. 2024; Zugal 2013). Beside self-assessment, which can be subjective, eye-tracking measures can provide reliable insights into cognitive load (Holmqvist et al. 2011; Figl et al. 2024; Abbad-Andaloussi et al. 2023a; Schreiber et al. 2024). Eye-tracking enables the analysis of fixation characteristics of readers (i.e., the amount of time the eye remains stationary at a specific position of the stimulus, e.g., a process model (Holmqvist et al. 2011)) to estimate their cognitive load (Holmqvist et al. 2011). The use of fixation features as indicators of cognitive load is grounded in the eye-mind hypothesis (Holmqvist et al. 2011), which postulates that the mind processes the content currently fixated by the eyes. Glöckner and Herbold (2008) extended this theory by suggesting that fixations lasting $\ge 250 ms$ signify mental processing, which can be linked to cognitive load.

While the eye–mind hypothesis was challenged, particularly because of its limitations in accounting for asynchronies between attention and eye movements, as well as its inability to capture the influence of peripheral vision on cognitive processing (Holmqvist et al. 2011), it continues to serve as a central assumption in research exploring how users engage with software artifacts. This is evident in a wide range of studies that adopt the hypothesis to examine various cognitive and behavioral aspects of users’ interactions with software artifacts (overview in Sharafi et al. (2015); Weber et al. (2021); Batista Duarte et al. (2021)).

To evaluate the effect of ambiguities on cognitive load, we adopt both the self-assessment of perceived difficulty measure and the eye-tracking measures reflecting cognitive load. For the former, we use a 5-point Likert scale questionnaire to capture perceived difficulty. For the latter, we use the mean number of fixations lasting $\ge 250 ms$ as an indicator of cognitive load. We hypothesize that both measures will exhibit significant increases when readers are confronted with ambiguous process models, reflecting the additional mental effort required to resolve the ambiguities.

*Comprehension.* In the cognitive science literature, very high levels of cognitive load can impair readers’ *performance*, particularly in terms of *task accuracy* (Veltman And Jansen 2005) and *response time* (Chen et al. 2016). Moving to the process model comprehension literature, *performance* is typically associated with *comprehension efficiency*, i.e., the ability to correctly interpret the given models (i.e., *comprehension (task) accuracy*, or shortly *comprehension accuracy*) within a limited time frame (i.e., *response time*) (Mendling et al. 2012; Turetken et al. 2020). Comprehension efficiency — as well as its individual components, comprehension accuracy and response time — has been widely used in the literature to assess the comprehension of process models (Figl 2017; Mendling And Strembeck 2008; Figl et al. 2024; Reinhartz-Berger et al. 2017; Reijers and Mendling 2010; Winter et al. 2023; Schreiber et al. 2024; Wang et al. 2022; Mendling et al. 2012; Turetken et al. 2020). Comprehension accuracy can be measured by comparing the reader’s task outcome (e.g., the response to a comprehension question) to a predefined ground truth (e.g., whether the response is correct). Response time, on the other hand, can be measured by capturing the time elapsed from the start to the completion of the task.

In our study, we adopt response time as the primary measure of comprehension efficiency, assuming that it will be reduced when readers are confronted with an ambiguous process model. We exclude comprehension accuracy from our analysis, as evaluating the correctness of responses to comprehension questions on ambiguous process models is not meaningful due to the potential for multiple valid interpretations.

*Visual Associations.* Eye-tracking is a powerful tool for tapping into readers’ visual associations, which refer to repeated shifts of attention between different model elements (Bera et al. 2019). Such associations are usually interpreted as an effort to mentally integrate visually associated elements (Bera et al. 2019). Therefore, through the investigation of visual associations using eye-tracking, we can gain insights into readers’ cognitive integration processes and analyze their underlying characteristics (Bera et al. 2019). When trying to disambiguate a process model, the search for additional information distributed across the model, combined with the continuous need to consolidate this information, is likely to result in a high frequency of visual associations. According to Bera et al. (2019), this pattern suggests an intensified demand for cognitive integration.

Visual associations can be quantified using the AOI (Area of Interest) Run Count (AOIRC) (Bera et al. 2019). The AOIRC has been widely adopted to capture visual associations and was proven to reflect the cognitive integration processes of readers (Bera et al. 2019). An AOI represents a specific region of a stimulus containing information pertinent to a particular analysis (Holmqvist et al. 2011). In the context of an eye-tracking study on process models, an AOI corresponds to a model element, such as an activity or a gateway (Holmqvist et al. 2011). The AOIRC measures the number of entries and exits to an AOI from other AOIs. As noted by Bera, Soffer and Parson in Bera et al. (2019), increased cognitive integration is associated with increased AOIRC values. Visual associations can also be examined through process maps (Gulden et al. 2020). When visual associations are low, the process maps are expected to illustrate a straightforward, sequential reading pattern. Conversely, when visual associations are high, the process maps are expected to illustrate a complex, non-linear reading pattern characterized by frequent transitions between fixated model elements.

We employ the AOIRC and process maps to analyze readers’ visual associations and cognitive integration. We anticipate an increase in the AOIRC when readers are confronted with ambiguous process models, as these models yield multiple interpretations and require greater effort to integrate the information into the readers’ mental schemas. This increased cognitive integration is expected to manifest in process maps that are more intricate and convoluted.

### Qualitative analysis of visual behavior during process comprehension

In this section, we present the background and related literature, focusing on qualitative analyses of readers’ visual behavior during process comprehension. This knowledge lays the groundwork for our subsequent qualitative investigation into how readers resolve ambiguities in process models (addressing RQ2, cf. Introduction section).

Eye-tracking provides various measures to evaluate the impact of different model-related factors on readers’ cognitive aspects (cf. Cognitive load, comprehension, and visual associations section). Besides, eye-tracking can be also used to discover new insights about readers’ behavior when engaging with process models. This is typically achieved through visualizations plotting eye-tracking data to illustrate readers’ behavior and help to identify reoccurring patterns. Eye-tracking visualizations are very diverse and can be used to emphasize different spatial and temporal aspects of readers’ visual behavior (cf. the overview in Holmqvist et al. (2011)).

In the context of process comprehension, authors have used various visualizations to analyze readers’ visual behavior (Winter et al. 2023; Djurica et al. 2023; Abbad-Andaloussi et al. 2021; Fındık-Coşkunçay and Çakır 2022). Some authors focused on the spatial characteristics of visual behavior through the projection of eye-tracking data on process artifacts (e.g., process models and decision tables used as stimuli in experiments). For instance, Winter et al. (2023) used heatmaps, focus maps, and scan-path projections to investigate the visual routines underlying the comprehension of process models. Similarly, Djurica et al. (2023) used scan-path projections to investigate readers’ search behavior when reading decision tables summarizing the control-flow of specific processes. Another group of researchers used visualizations that emphasize the temporal aspects of visual behavior. Therein, for instance, both Fındık-Coşkunçay and Çakır (2022) and Abbad-Andaloussi et al. (2021) used scarf plots to analyze the evolution of readers’ engagement with process models over time. These plots highlighted the sequential order in which different parts of the models were visited and the duration of these visits. Additionally, they illustrated the interplay between various parts of the models and how specific parts were revisited over time. Fındık-Coşkunçay and Çakır (2022) investigation was about joint visual attention in collaborative process comprehension, while the study by Abbad-Andaloussi et al. (2021) was about identifying the strategies used by IT specialists and domain experts to understand hybrid process models where the models are enriched with textual annotations explaining process activities and guided simulations illustrating the models semantics.

While eye-tracking data is effective for tracing readers’ visual behaviors, using it alone may not provide explanations for their behaviors or help establishing connections to specific cognitive processes or task-solving strategies (Holmqvist et al. 2011). To address this limitation, it is generally recommended to complement eye-tracking data with verbal data, which can be collected through concurrent or retrospective think-aloud methods (Holmqvist et al. 2011). In concurrent think-aloud, participants verbalize their thoughts in real-time as they perform a task. In retrospective think-aloud, participants reflect on their actions and decisions after completing the task (Holmqvist et al. 2011). Once verbal data is collected, it is analyzed to extract meaningful insights that contextualize the visual behavior observed with eye-tracking (Holmqvist et al. 2011). This combined approach enables researchers to make more confident inferences about readers’ cognitive processes and the strategies they use to solve the given tasks (Holmqvist et al. 2011).

In the process model comprehension literature, verbal data collected through concurrent think-aloud has been, for instance, combined with eye-tracking data by Bera et al. (2019), to interpret readers’ visual association behavior and link it to cognitive integration. Similarly, verbal data collected retrospectively has been combined with eye-tracking data in the aforementioned study of Abbad-Andaloussi et al. (2021) to confirm the strategies underlying the comprehension of hybrid process models.

## Research method

To explore the cognitive effects of ambiguity on process model readers, as well as their behavior when resolving ambiguity, we conducted an eye-tracking study, adhering to the guidelines outlined in Ralph et al. (2021). This section reports on the study design, execution, and data analysis.

### Study design

#### Research Model to Answer RQ1

**Fig. 3:**
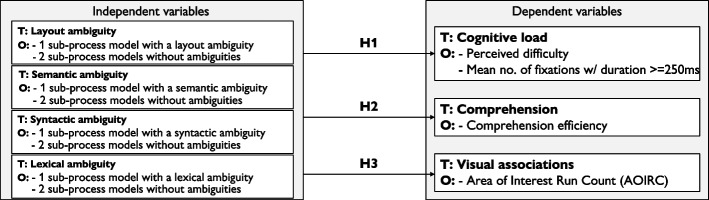
Research model to answer RQ1. T = theoretical construct; O = operationalization of the construct

The objective of this study with respect to RQ1 is to investigate how different types of ambiguity in process models affect model readers’ cognitive load, comprehension and visual associations. To achieve this, we adopt a within-subject design, guided by the research model illustrated in Fig. 3. Each type of ambiguity (i.e., *layout, semantic, syntactic* or *lexical*), is treated as a theoretical construct on the independent variables side, and is examined separately for its influence on the dependent variables (i.e., *cognitive load*, *comprehension*, and *visual associations*), which in Fig. 3 are denoted as theoretical constructs on the dependent variables side.

At the independent variables side, each *type of ambiguity* (i.e., a factor) is defined with two factor levels: *no ambiguity* and *ambiguity*. In our study design, these levels are operationalized within a process model consisting of two sub-processes *without* ambiguities and one sub-process *with* an ambiguity of that specific type. At the dependent variables side, *cognitive load* is operationalized through *readers’ self assessment of perceived difficulty* and the *mean number of fixations associated with mental processing* (i.e., duration $\ge 250ms$, cf. Cognitive load, comprehension, and visual associations section). *Comprehension* is operationalized through *comprehension efficiency* (i.e., time required to complete a task, cf. 2.2 section). Finally, *visual associations* are operationalized using the *AOIRC measure* (cf. Cognitive load, comprehension, and visual associations section). All these measures are collected at the sub-process level.

Our within-subject design enables a pairwise within-subject comparison between the two factor levels (*no ambiguity* and *ambiguity*) of each independent variable with respect to the dependent variables. Building on the theoretical foundations presented in Cognitive load, comprehension, and visual associations section on the effects of ambiguity on readers’ cognitive load, comprehension, and visual associations, we propose the following hypotheses ${\textbf {H1}}$–${\textbf {H3}}$. These hypotheses apply to each of the four types of ambiguity. For brevity, when considering a specific ambiguity, we use the notation ${\textbf {Hx}}_{prag}$, ${\textbf {Hx}}_{sem}$, ${\textbf {Hx}}_{syn}$, and ${\textbf {Hx}}_{lex}$, where ${\textbf {Hx}}$ corresponds to the hypothesis number:

- ${\textbf {H1:}}$ A sub-process model with ambiguity yields a higher cognitive load compared to a sub-process model without ambiguity.
- ${\textbf {H2:}}$ A sub-process model with ambiguity yields lower comprehension compared to a sub-process model without ambiguity.
- ${\textbf {H3:}}$ A sub-process model with ambiguity yields higher visual associations than a sub-process model without ambiguity.

#### Research Model to Answer RQ2

The goal of our study with respect to RQ2 is to investigate the visual behaviors exhibited by model readers when resolving ambiguities in process models. To this end, we adopt a qualitative study design structured around the Goal, Question, Metric (GQM) template (Caldiera And Rombach 1994), which is depicted in Table 1. Our investigation is based on qualitative codes that emerged from the analysis of behavioral data collected through eye-tracking technology and retrospective think-aloud. These codes reflect behavioral patterns uncovering how model readers resolve ambiguities.

**Table 1:** GQM model to answer RQ2 describing our goal, research question and used indicators

Goal	Purpose	Explore
	Issue	how model readers react to process model ambiguity
	Object	in terms of patterns of visual behavior
	Viewpoint	from the viewpoint of BPM practitioners with different expertise
Question	RQ2	What patterns of visual behavior do model readers exhibit when resolving ambiguities in process models?
Indicators		Qualitative codes emerging from the eye-tracking and think-aloud data of participants performing process comprehension tasks

#### Material

For the study material, we designed 13 process models representing manufacturing processes using Camunda Modeler^2^. Each model was designed to include exactly one ambiguity, ensuring that no two models shared the same ambiguity. One model was used for a familiarization task to allow participants to become accustomed to the experiment task and ask questions if needed. The remaining 12 models were divided as follows. Three models were affected by one layout ambiguity each, three by one semantic ambiguity each, three by one syntactic ambiguity each, and three by one lexical ambiguity each. Building on prior literature that proposed several modeling guidelines aimed at avoiding ambiguity in process models (cf. Corradini et al. (2018); Mendling et al. (2010c)), we deliberately violated specific guidelines, targeting specific model elements, e.g., a gateway or an activity label. These violations rendered the model element ambiguous. For example, to violate guideline *35: Labeling AND-gateways* from Corradini et al. (2018), we added labels to an AND-split gateway, thereby introducing ambiguity. As these violated guidelines are derived from multiple studies (Corradini et al. 2018; Mendling et al. 2010c), we consider this method suitable for generating ambiguities that are noticeable by the study participants. Moreover, we designed the process models through multiple rounds of internal validation and iterative refinement until reaching consensus among all authors that the models embodied the intended ambiguities. This choice was deliberately made to maximize the likelihood that the introduced ambiguity, which is inherently subjective, would lead to multiple valid interpretations among study participants. After the internal validation rounds, we conducted pilot tests to confirm that no introduced ambiguity went unnoticed.

Following our within-subject study design, each process model consisted of a sequence of three sub-processes. Ambiguity was introduced exclusively in one of the sub-processes (i.e., the second), to enable a direct comparison of cognitive and behavioral measures associated with the analysis of sub-processes with and without ambiguity. To minimize confounding factors related to model size and complexity, all sub-processes were designed to be uniform in terms of size and complexity. Specifically, each sub-process had 10 activities, 1 intermediate event, and 3 pairs of split-join gateways. Their complexity was also standardized across 17 complexity metrics. Since domain knowledge could act as a confounding factor, allowing individual participants to use their expertise to resolve the ambiguities (Wohlin et al. 2012), activity labels were randomized to mitigate this effect. The labels followed a verb-object style, combining a random verb from the manufacturing domain with the term *“piece”* and a random three-letter string (e.g., *Extrude piece TRS*). The verbs were chosen to denote generic transformations of work pieces that can be combined arbitrarily in a sequence, thereby always yielding plausible processes. This approach ensured preventing the generation of nonsensical models, while remaining neutral with respect to participants’ domain knowledge.

We designed one task per process model, with each task tailored to the specific ambiguity affecting the model. For tasks involving layout, semantic, and syntactic ambiguity, the task asked the question *“Name a valid trace for the process, assuming that the temperature T=200”*. This question was designed to require the participants to sequentially analyze the control flow of the entire model. Specifying a temperature value (i.e., *T=200*) ensured that the participants focused on identifying a single, specific trace. Since layout, semantic, and syntactic ambiguities affect the control flow, this task design guaranteed that participants encountered and had to interpret the associated ambiguity to solve the task. For example, one model used for this task included an AND-split gateway whose outgoing control flow edges were annotated with conditions. To answer the question, participants had to read the gateway and the conditions, interpreting them to name a trace. On the other hand, for tasks involving lexical ambiguity, where the control flow is not relevant, the questions emphasized the lexicon of the labels. Here, we formulated questions such as *“How many pieces are moved?”* or *“How many pieces are tested?”* to encourage participants to carefully read and interpret all activity labels in the model, including the ambiguous ones. For example, the model associated with the question *“How many pieces are tested?”* included an activity labeled *“Design test of piece TRS”*, which could be interpreted in multiple ways (e.g., designing the test or testing the design of a piece), leading to different answers.

#### Participants

In total, 44 participants from St.Gallen University (20 participants) and Karlsruhe Institute of Technology (24) were recruited for the experiment (cf. Table 2). The participants were representative of the age groups 20–30, 30–40, 40–50, and 50+; the majority of the participants (64%) fell into the 20–30 age group. The set of participants included 17 Bachelor’s and Master’s students, 18 researchers at the pre- and post-doctoral level, 4 IT employees, and 5 students also working as IT employees. The participants had a BPM expertise ranging from 0 to 25 years; they all had a basic understanding of BPMN or similar modeling notations. The within-subject experiment design prevents the participants’ heterogeneity from affecting the testing of our hypotheses.

**Table 2:** Demographic data of the participants

Demographics	Groups	Count	Percentage (%)
Age group	20-30	29	0.66
	30-40	11	0.25
	40-50	3	0.07
	> 50	1	0.02
Profession	Researcher	18	0.41
	Student	17	0.39
	Working student	5	0.11
	IT Industry	4	0.09
Familiarity with BPMN process models	1 (strongly disagree)	6	0.14
	2	8	0.18
	3	4	0.09
	4 (neutral)	3	0.07
	5	12	0.27
	6	8	0.18
	7 (strongly agree)	2	0.05

### Experiment procedure

**Fig. 4:**
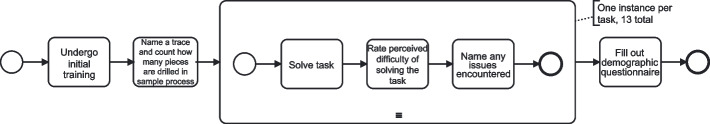
Experiment procedure

The experiment was conducted as individual sessions lasting approximately one hour in a controlled lab environment. The experiment procedure is illustrated in the BPMN diagram in Fig. 4. Initially, participants underwent a training on the basic BPMN elements present in the models used in the study. To prevent bias, participants were not informed about the study goals or the topic of ambiguity in process models. Following this, they were asked to perform two practice tasks: naming a valid trace and counting how many pieces are manipulated in an example process. These tasks were conducted to ensure that participants understood the types of questions used in the experiment (cf. Study design section). During this phase, participants could ask questions and receive feedback. After the training, the actual experiment began with a familiarization task. This task allowed participants to get accustomed with the interface and task format without influencing the study results, as the outcomes of this task were excluded from the subsequent data analysis. To address potential learning and fatigue effects, the presentation order of the remaining tasks was randomized. This randomization ensured that learning effects and participant fatigue were distributed evenly across the different experiment tasks. After completing each task, participants rated the perceived difficulty of solving the task for each sub-process using a Likert scale. These ratings were used to compute the perceived difficulty measure of cognitive load (cf. Study design section). Participants were also asked retrospectively to state whether they encountered any issues while solving the task and to describe how they overcame them. This was used to inform us whether they had noticed the ambiguities in the process models and how they resolved them. We used this information in the data analysis to consider only tasks where ambiguity was noticed and to qualitatively analyze participants’ behavior when resolving ambiguities (cf. Data collection and analysis section).

### Data collection and analysis

The data was gathered using EyeMind (Abbad-Andaloussi et al. 2023b), which is an eye-tracking tool designed for capturing eye-tracking data on process models displayed within an interactive editor. This tool allows users to seamlessly navigate various parts of a model and explore its sub-processes. A significant advantage of EyeMind is its ability to support dynamic eye-tracking stimuli, enabling users to freely browse through different views, scroll, and zoom in various parts of the stimulus (Abbad-Andaloussi et al. 2023b; Holmqvist et al. 2011). Conducting experiments with dynamic stimuli is recognized as a complex and time-intensive process (Abbad-Andaloussi et al. 2023b; Holmqvist et al. 2011). Consequently, researchers often rely on static stimuli, using small, non-interactive process models presented as images. While simpler to implement, this approach does not accurately represent the complexity and usability of real-world process models, limiting the ecological validity (i.e., ability to generalize findings) (Abbad-Andaloussi et al. 2023b; Holmqvist et al. 2011). To overcome the limitations of relying on static stimuli and better reflect the size and complexity of real-world models, we selected EyeMind. To the best of our knowledge, it is the only tool capable of providing this functionality for process models.

After data collection, we selected the trials^3^ in which participants correctly identified the ambiguities embedded in the models. This selection was based on the assumption that non-identified ambiguities would have no effect on participants’ cognitive and behavioral responses. Furthermore, this selection was based on our goal to investigate the effects of *recognized* ambiguity as the enabler of the cognitive and behavioral aspects that we set to study. This is because we could not measure the effects of what was not perceived as ambiguous and did not result in such effects. Ambiguities were identified by the participants in 342 trials out of 528 trials (derived from 44 participants completing 12 tasks, excluding the familiarization task). To determine these trials, participants who stated having encountered issues in solving a task were asked to navigate through the sub-process models to show us the specific fragment responsible for their difficulties and explain us the respective reasons. We selected the trials in which the indicated fragment matches with the ambiguity and the explanation could be associated with the ambiguity. In a limited number of cases, participants noted encountering additional issues that were not related to ambiguous fragments, such as finding an xor-gateway condition not immediately clear at first sight. In these cases, there were no unintended ambiguities discovered by the participants, but only issues beyond ambiguity. Since these issues were not related to ambiguity and our study focuses on the effects of ambiguity, we did not include the respective data in our analysis. To address RQ1 (cf. Introduction section), we calculated the measures corresponding to the constructs on the dependent variables side of the research model illustrated in Fig. 3. We conducted these calculations at the level of each sub-process. Since participants were assigned three tasks per ambiguity type (cf. Study design section), three data points were collected per factor level and participant. To mitigate inter-dependencies among data points, we computed the mean value of each measure at each factor level for each participant. We computed both descriptive and inferential statistics, as reported in Table 3. Descriptive statistics facilitated *pairwise* within-subject comparisons between the mean values of each measure across the factor levels: *ambiguity* and *no ambiguity* for each ambiguity type. To determine the statistical significance of the observed differences, we used the Wilcoxon Signed-Rank inferential test (Wilcoxon 1945), which is suitable for pairwise within-subject comparisons and does not impose assumptions on the normal distribution of the data.

**Table 3:** Descriptive and inferential statistics

A.	H.	Measure	Descriptive		Inferential
			No Ambiguity	Ambiguity	*p*-value
			Mean	Mean	
**Layout A.**	**H1**	**Cognitive Load**			
		Perceived Difficulty	1.038	**3.331**	$\varvec{<}$**.001**
		Fixations $>=250$ ms	32.754	**74.508**	**<.001**
	**H2**	**Comprehension**			
		Comprehension Efficiency	27198.497	**51667.651**	$\varvec{<}$**.001**
	**H3**	**Visual Associations**			
		AOI Run Count	21.159	**46.357**	$\varvec{<}$**.001**
**Semantic A.**	**H1**	**Cognitive Load**			
		Perceived Difficulty	0.893	**2.240**	$\varvec{<}$**.001**
		Fixations $>=250$ ms	28.354	**41.547**	$\varvec{<}$**.001**
	**H2**	**Comprehension**			
		Comprehension Efficiency	25809.034	**39514.319**	$\varvec{<}$**.001**
	**H3**	**Visual Associations**			
		AOI Run Count	20.067	**34.573**	$\varvec{<}$**.001**
**Syntactic A.**	**H1**	**Cognitive Load**			
		Perceived Difficulty	0.973	**2.217**	$\varvec{<}$**.001**
		Fixations $>=250$ ms	25.212	**34.308**	$\varvec{<}$**.001**
	**H2**	**Comprehension**			
		Comprehension Efficiency	26414.001	**32977.135**	$\varvec{<}$**.001**
	**H3**	**Visual Associations**			
		AOI Run Count	19.151	**27.489**	$\varvec{<}$**.001**
**Lexical A.**	**H1**	**Cognitive Load**			
		Perceived Difficulty	0.786	**1.976**	$\varvec{<}$**.001**
		Fixations $>=250$ ms	**21.610**	21.463	0.634
	**H2**	**Comprehension**			
		Comprehension Efficiency	16258.680	**19436.565**	**0.019**
	**H3**	**Visual Associations**			
		AOI Run Count	18.086	**22.610**	**0.008**

Comprehension Efficiency unit: milliseconds. *Note:*$p<$0.05 informs that the pairwise difference of means between the no ambiguity and ambiguity levels is significant

**Fig. 5:**
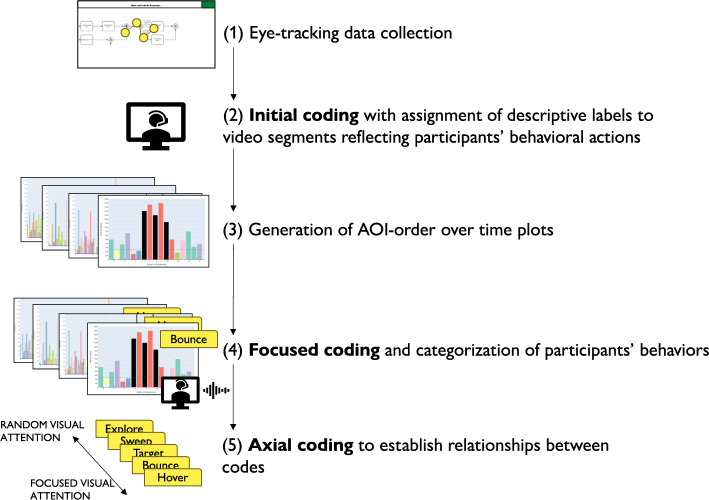
Overview of the qualitative exploratory analysis

Besides validating the impact of ambiguity, we conducted a qualitative exploratory analysis, using the participants’ eye tracking and think-aloud data (cf. overview in Fig. 5). Herein, the aim was to investigate their behavior when resolving ambiguity (RQ2, cf. Introduction section). Specifically, we used a qualitative coding approach from grounded theory (Charmaz 2006; Bryant and Charmaz 2019) to identify common behaviors adopted by the participants. The codes describing visual behavior patterns were developed based on preliminary observations of the video recordings (of approximately 40 minutes each) of a sample of 23% of the study participants. An *initial coding* (Bryant and Charmaz 2019) phase was conducted on these videos to identify emerging visual behaviors. Herein, we assigned descriptive labels to video segments, reflecting the participants’ behavioral actions (e.g., fixating on a specific model element for a long period of time or shifting rapidly across the model), without making assumptions about the meaning of these actions. This phase allowed us to remain open to all possible interpretations and to capture the full range of behaviors participants exhibited when engaging with ambiguous process models. Afterward, we generated *AOIs-order over time* plots of all the trials in which ambiguities were noticed.

**Fig. 6:**
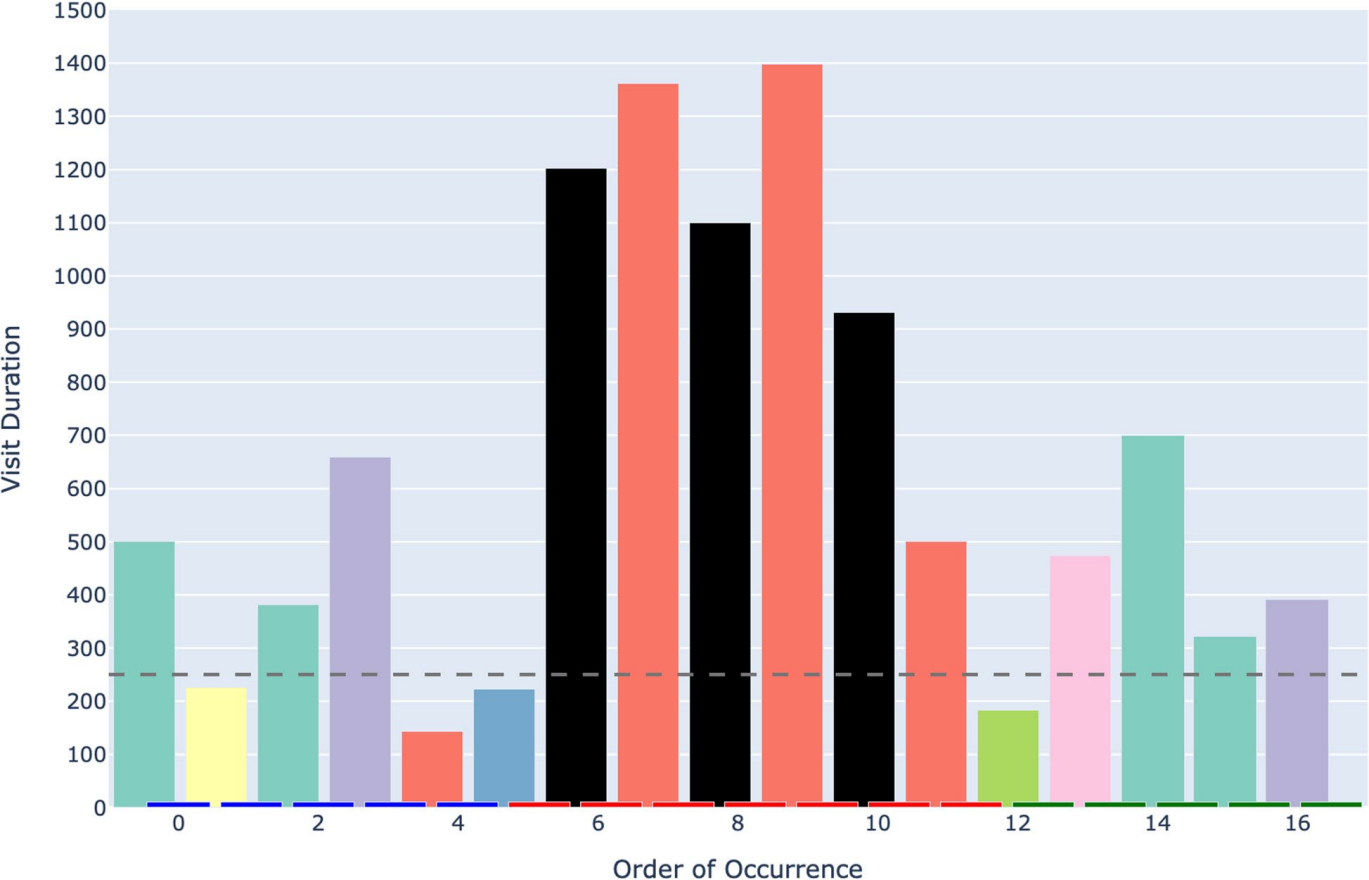
Example of a *simplified* AOIs-order over time plot

**Fig. 7:**
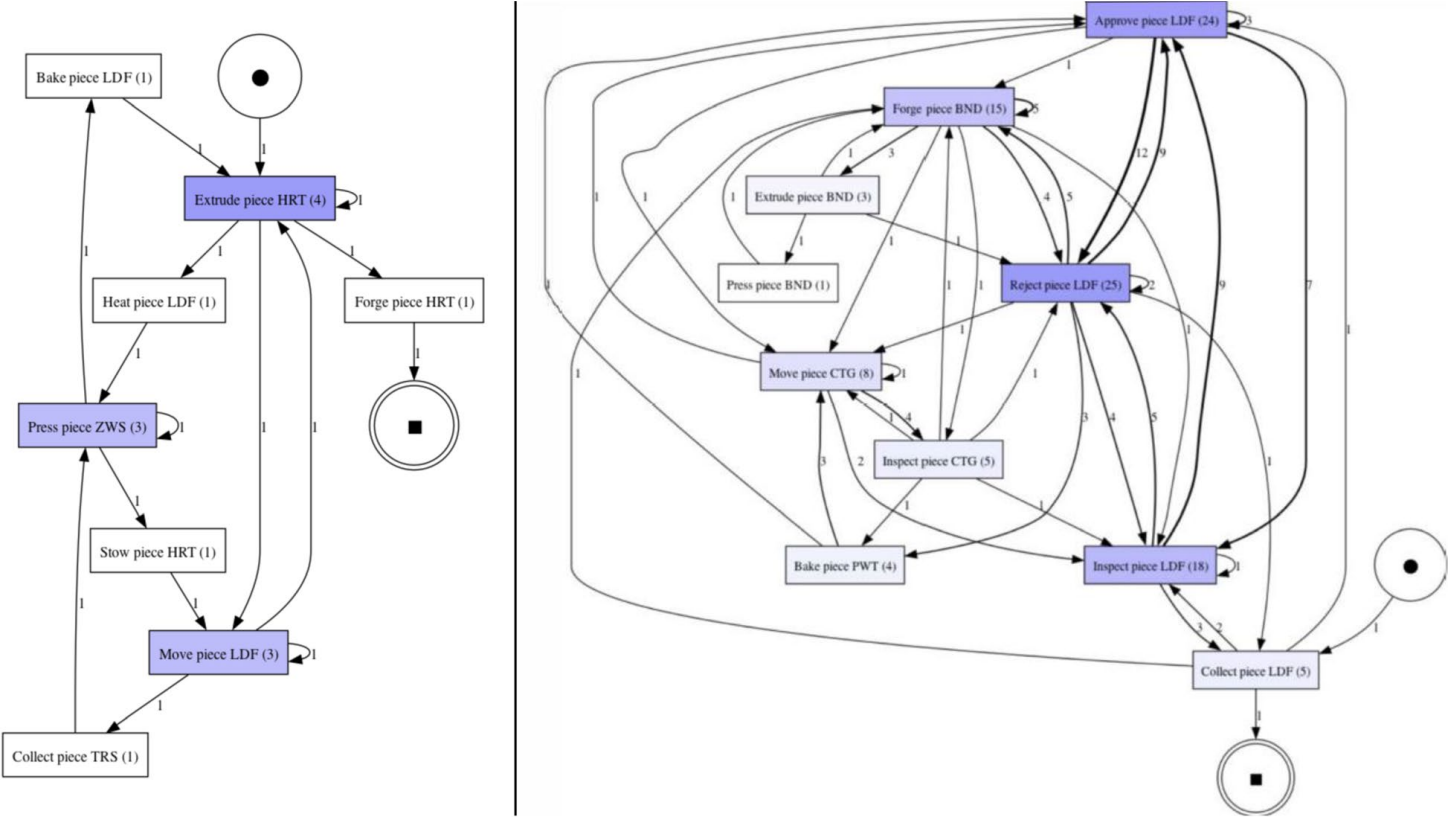
Process maps comparing the visual associations of a participant (SP7) when reading a sub-process without (left) and with (right) a semantic ambiguity. A higher resolution of this figure is available in the online appendix. The circle with a dot inside denotes the process start, the double circle with a square inside denotes the process end. Rectangles refer to visits to the different process model activities; edges refer to the transitions for visiting one activity from another. The color scale of the rectangles refers to the absolute visit frequency to an activity; the thickness and labels on the edges refer to the absolute transition frequency, resp. the number of transitions between each pair of activities

**Fig. 8:**
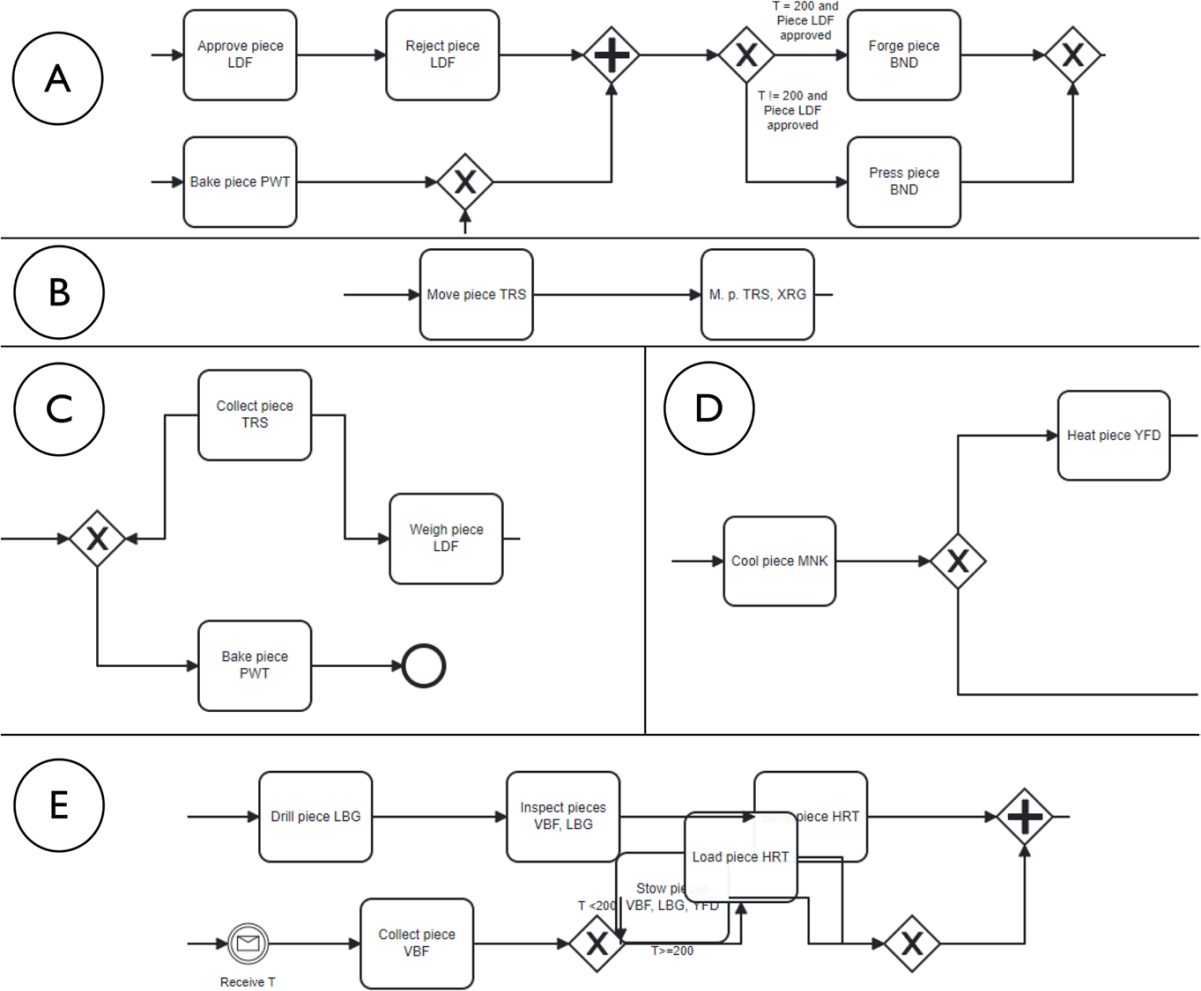
Excerpts from ambiguous process models showing the ambiguous model fragments. The complete models are available in the online appendix

Figure 6 depicts an example of an AOIs-order over time plot. This plot visualizes how a participant’s attention shifts across different elements (represented as AOIs) of a process model while performing a task. The bars on the X-axis show the AOIs visited by the participant, sorted by their order of occurrence in time. This temporal perspective reflects the sequence of process model elements that the participant visited during the task. The black AOIs refer to the ambiguous elements of the process model, while the remaining AOIs refer to the non-ambiguous elements of the model. This latter set of AOIs have different colors, allowing, in turn, to identify those that were visited several times by the participant. This color coding helps visualize how the participant shifted between ambiguous and non-ambiguous elements of the model. The X-axis is additionally decorated with horizontal blocks, colored in blue, red, or green to infer respectively whether the visited AOI belongs to the first, second, or third sub-process of our model. As mentioned in Study design section, the second sub-process was the one incorporating an ambiguity. On the Y-axis, the height of the bars defines the duration of each AOI visit (in milliseconds). Moreover, we have set 250*ms* as a threshold on the Y-axis, allowing to distinguish the visits reflecting mental processing (Glöckner And Herbold 2008). Projecting visit duration onto the Y-axis facilitates the comparison of how long participants engaged with ambiguous versus non-ambiguous model elements, and helps to identify visits likely associated with mental processing. All in all, the AOIs-order over time plot enables the analysis of participants’ visual behavior by revealing the sequence in which process model elements are visited (via the X-axis), the duration of each visit to a specific element (via the Y-axis), and which visits are likely to involve mental processing (Figs. 7 and 8).

**Fig. 9:**
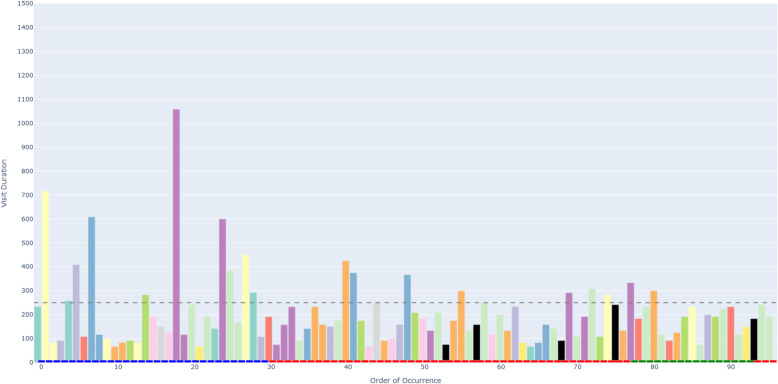
AOIs-order over time plot showing the *Sweep* visual behavior of participant SP7 while trying to resolve an ambiguity, visible in the sequence of short visits ($< 250ms$) to multiple process elements shown in different colors between the first and the last visits to the ambiguity (shown in black)

**Fig. 10:**
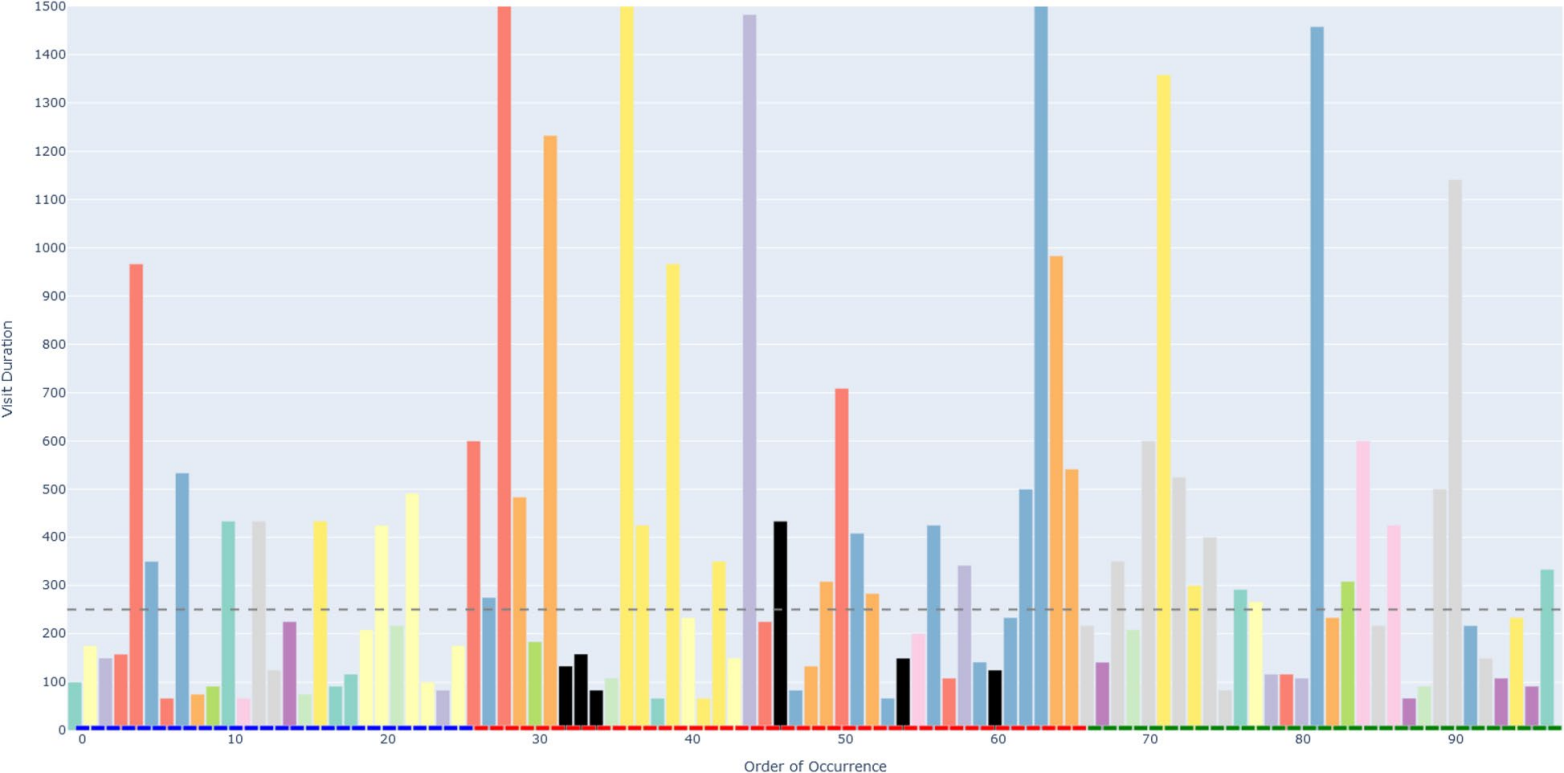
AOIs-order over time plot showing the *Explore* visual behavior of participant SP6 while trying to resolve an ambiguity, visible in the sequence of long visits ($\ge 250ms$) to multiple process elements shown in different colors between the first and the last visits to the ambiguity (shown in black)

**Fig. 11:**
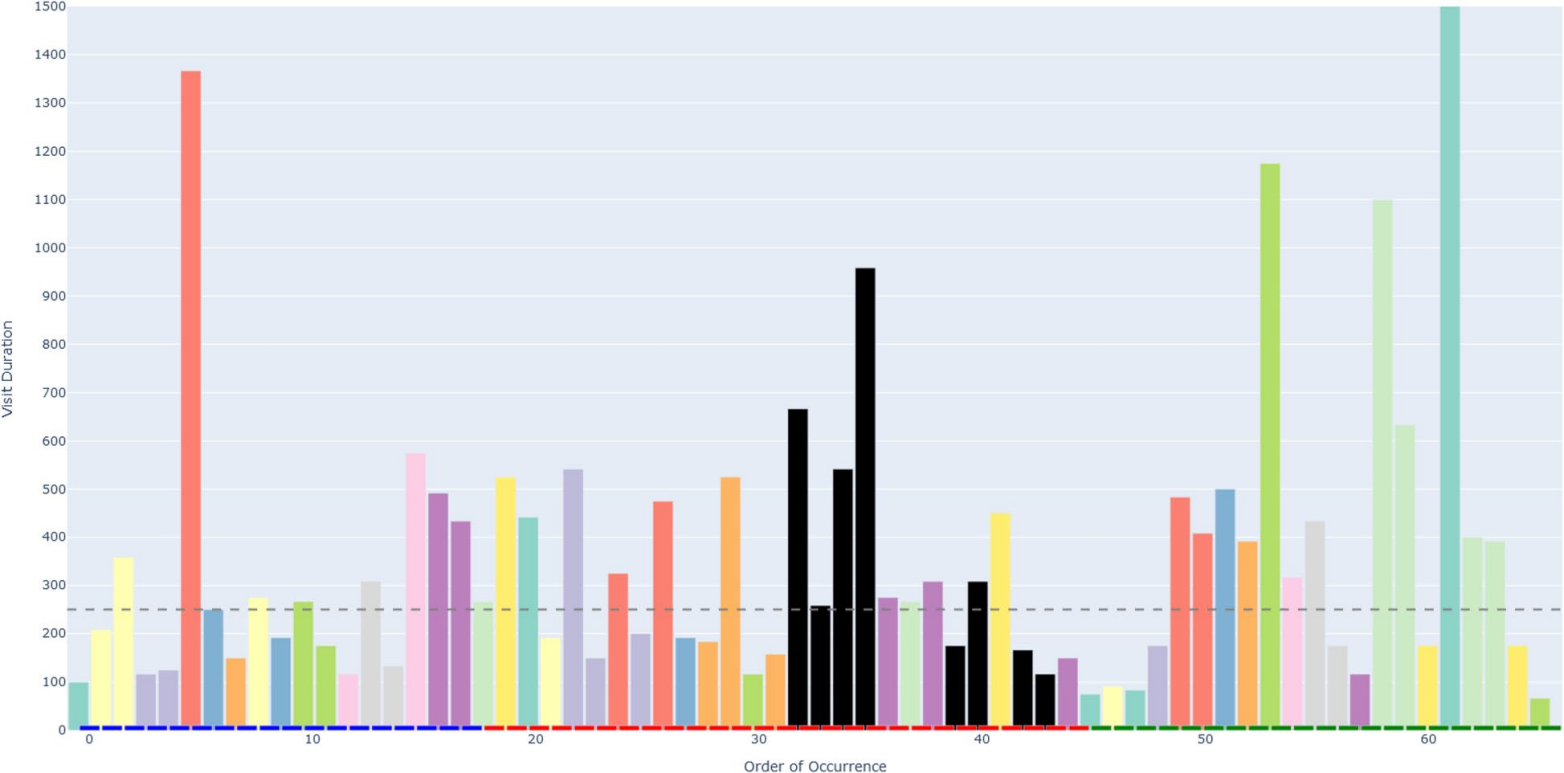
AOIs-order over time plot showing the *Target* visual behavior of participant KP23 while trying to resolve an ambiguity, visible in the sequence of long visits ($\ge 250ms$) to the few process elements shown in yellow, green and violet between the first and the last visits to the ambiguity (shown in black)

**Fig. 12:**
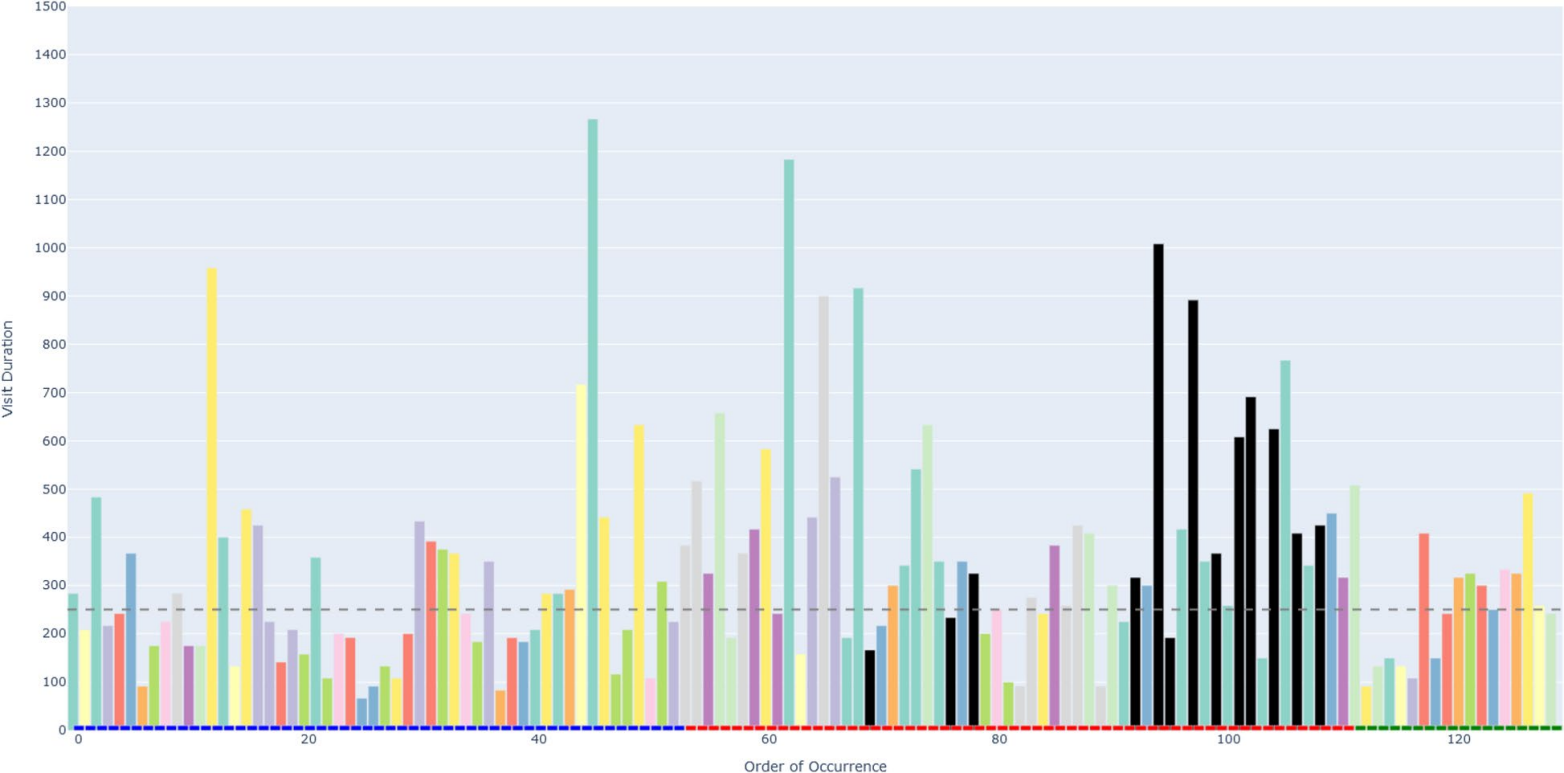
AOIs-order over time plot showing the *Bounce* visual behavior of participant SP20 while trying to resolve an ambiguity, visible in the alternating visits to the ambiguous process elements (shown in black) and another process element (shown in turquoise)

**Fig. 13:**
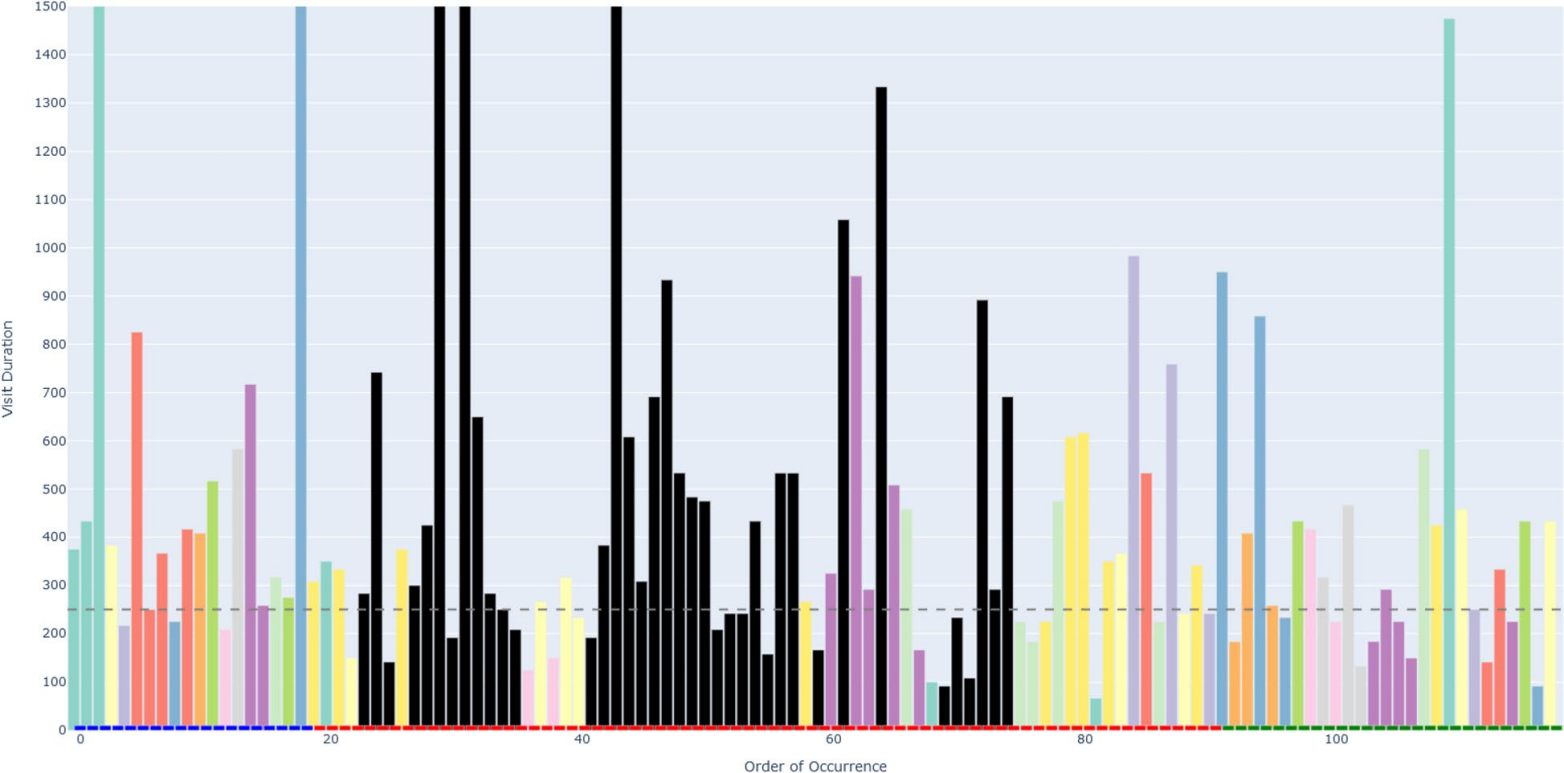
AOIs-order over time plot showing the *Hover* visual behavior of participant SP9 while trying to resolve an ambiguity, visible in the (mostly) uninterrupted sequence of multiple long visits to the ambiguous process elements (shown in black)

**Fig. 14:**
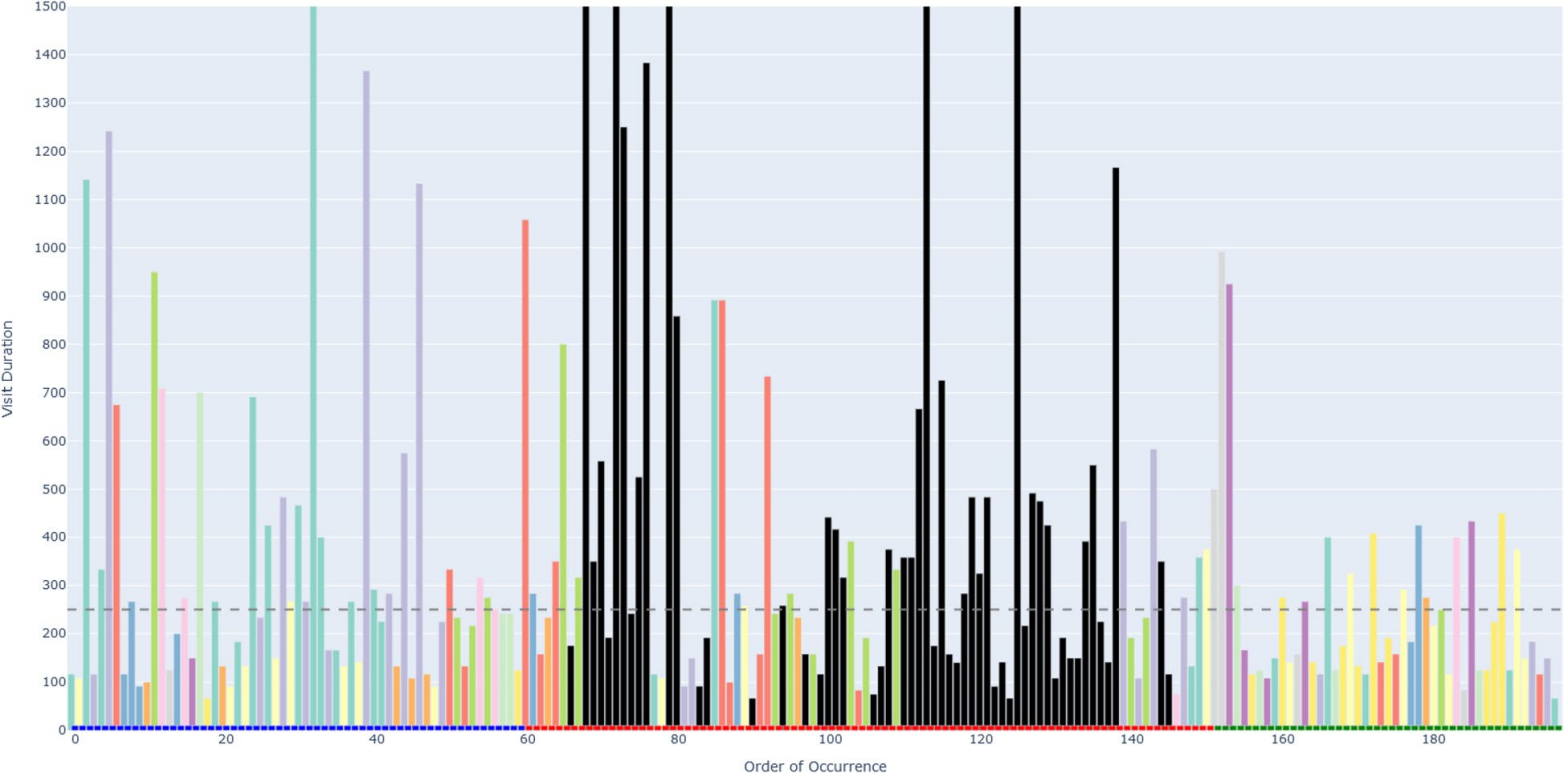
AOIs-order over time plot showing the interleaved visual behavior between *Hover* and *Target* by SP3 while trying to resolve an ambiguity

Following the generation of the AOIs-order over time plots, we conducted focused coding (Bryant and Charmaz 2019) on all these plots through the observation and coding of participants’ behavioral patterns, which were aligned with those observed in the videos. In this phase, we systematically categorized recurring behaviors by grouping similar initial codes into more abstract and conceptually meaningful codes. For instance, shifting short visits all over the process model elements without focusing on specific elements was coded as *Sweep*, while shifting long visits across a large area of the model covering several process elements was coded as *Explore*. When long visits occurred between a limited number of specific process elements, we assigned the code *Target*. Back-and-forth transitions between the ambiguity and another specific process element were captured under the code *Bounce*, whereas mostly uninterrupted sequences of long visits to the ambiguous process elements themselves were labeled *Hover*. These focused codes allowed us to infer the common strategies participants used when attempting to resolve ambiguities. To ensure the robustness of our codes, the coding was done by one author and subsequently reviewed by another author; in case of disagreements, the authors discussed the code until convergence. This coding strategy (aligned with prior literature, e.g., Jayaraman et al. (2024)) was intentionally chosen over independent coding considering the inherent complexity of the AOIs-order over time plots, whose visual analysis requires collaborative effort, iterative back-and-forth discussions, and careful inspection with multiple pairs of eyes to discern the behavioral patterns (cf. Figs. 9, 10, 11, 12, 13 and 14). Moreover, to ensure the validity of our patterns identification, we incorporated as supplementary evidence the verbal data obtained by asking retrospectively at the end of each task which strategies the participants employed to overcome any difficulties encountered. To do this, we listened to the audio track of the video recordings of the data collection sessions, timestamping the periods where relevant answers were provided, and taking note of the answers. This allowed us to triangulate the coded behaviors with the verbal insights provided by the participants. Finally, we applied axial coding to establish relationships between our codes (Bryant and Charmaz 2019). In doing so, we organized the focused codes into a scale of attention focus, arranging the identified behaviors according to their associated levels of attention, ranging from random attention (e.g., *Sweep*) to focused attention (e.g., *Hover*). This last coding phase was conducted collaboratively between the authors.

### Data availability and reproducibility

To ensure transparency, reproducibility, and replicability, we provide an online appendix, which includes:

- the full set of process models used in the tasks of the study;
- a comprehensive report detailing the complexity metrics of the process models;
- list of the experiment tasks with the specific guideline violations and the ambiguities these violations introduced;
- demographic information of the participants;
- a higher-resolution version of Figs. 4, 7 and 8;
- process maps illustrating the participants’ visual associations;
- all AOIs-order over time plots derived from the eye-tracking data;
- the qualitative codes with the behaviors observed;
- the Python notebook used for data analysis, along with the corresponding results.

The online appendix can be accessed at https://doi.org/10.5281/zenodo.16738478.

## Findings

In this section, we present our findings organized by research question.

### RQ1. How do different ambiguities affect model readers’ cognitive load, comprehension and visual associations?

**Cognitive load.** Cognitive load was measured using perceived difficulty and mean number of fixations with duration $\ge 250ms$, collected at the level of both ambiguous and non-ambiguous sub-process models (cf. Cognitive load, comprehension, and visual associations section). As illustrated in Table 3, ambiguous sub-process models resulted in higher perceived difficulty compared to non-ambiguous sub-process models across all tasks. The reported *p*-values confirm the statistical significance of these differences. For the mean number of fixations $\ge 250ms$, significant differences were observed for layout, semantic, and syntactic ambiguity. However, for tasks involving lexical ambiguity, no significant differences were detected, as indicated by a *p*-value of 0.634. These results confirm hypotheses ${\textbf {H1}}_{prag}$, ${\textbf {H1}}_{sem}$, and ${\textbf {H1}}_{syn}$ while rejecting ${\textbf {H1}}_{lex}$.

**Comprehension.** Comprehension efficiency was measured by measuring the time required to complete a task (cf. Cognitive load, comprehension, and visual associations section). Table 3 presents the mean completion times for both non-ambiguous and ambiguous sub-process models. Across all tasks, the time required for ambiguous sub-process models was higher than for non-ambiguous models. The statistical significance of these differences was confirmed by the *p*-values, thus confirming hypotheses ${\textbf {H2}}_{prag}$, ${\textbf {H2}}_{sem}$, ${\textbf {H2}}_{syn}$, and ${\textbf {H2}}_{lex}$.

**Visual Associations.** To explore how different ambiguities influence model readers’ visual associations, we measured the AOIRC (cf. Cognitive load, comprehension, and visual associations section). As illustrated in Table 3, the AOIRC relative to ambiguous sub-process models was higher compared to non-ambiguous sub-process models across all tasks. These differences were statistically significant, as indicated by the *p*-values, thereby confirming hypotheses ${\textbf {H3}}_{prag}$, ${\textbf {H3}}_{sem}$, ${\textbf {H3}}_{syn}$, and ${\textbf {H3}}_{lex}$. Figure 7 present process maps that illustrate these differences, showcasing the stark contrast in visual associations (shifts of attention between model elements) when a reader analyzes a non-ambiguous sub-process model (few shifts of attention) versus an ambiguous one (many shifts of attention) within the same task. Similar process maps for all tasks and participants are included in the online appendix (cf. Data availability and reproducibility section).

### RQ2. What patterns of visual behavior do model readers exhibit when resolving ambiguities in process models?

Following the qualitative coding approach (cf. Data collection and analysis section), we identified five codes associated with distinct visual behavior patterns, which we named as follows: *Sweep*, *Explore*, *Target*, *Bounce*, and *Hover*. In the following, we introduce these behavior patterns and give representative examples from our study illustrating them. For more detailed insights from all the study participants, we refer the reader to the online appendix (cf. Data availability and reproducibility section).

***Sweep*** corresponds to short visits ($< 250ms$) all over the process model without focusing on any specific process elements. For example, participant SP7 exhibited this visual behavior pattern when confronted with a process model in which ambiguity was due to an XOR gateway without any conditions specified (the ambiguous model fragment is depicted in Fig. 8, D). This gateway could be interpreted as an incorrectly modeled AND gateway, or as an XOR gateway whose condition is implicit in the process context. SP7’s visual behavior in response to the ambiguity is visualized in the AOIs-order over time plot shown in Fig. 9. The plot shows multiple mostly short visits to different process elements between the first and the last visits to an ambiguous process model element. When asked about the approach taken to overcome the ambiguity, SP7 responded “*Here I didn’t know what to do [...] I wouldn’t know where to route potentially and I wasn’t sure whether I could have continued with a potentially correct trace. So I didn’t know whether I should have continued both traces as a valid possibility.*” Here, SP7 stated being unable to determine what could be done in order to overcome the ambiguity. Our analysis of the think-aloud data suggests that this behavior relates to a difficulty in determining where to look for relevant information to resolve the ambiguity, translating in turn into a sweep behavior characterized by short visits all over the model. This can happen, for example, when it is unclear where to find clues helping to interpret an ambiguous XOR condition. Looking at our entire dataset, *Sweep* behavior, with similar characteristics, occurred in 43 trials.

***Explore*** corresponds to long visits ($\ge 250ms$) across a large area of the process model, covering several process elements. For example, participant SP6 exhibited this visual behavior pattern when confronted with a conditional flow in which the decision on the continuation is based on two activities with opposite semantics (“Approve piece LDF” and “Reject piece LDF”), executed in sequence before the split, making the semantics of their execution, hence the decision, unclear (the ambiguous model fragment is depicted in Fig. 8, A). The visual behavior of SP6 in response to the ambiguity is visualized in the plot in Fig. 10. The plot shows multiple long visits to several process elements between the first and the last visits to the ambiguity. In relation to overcoming the ambiguity, SP6 stated “*The condition in front of the ‘Forge piece BND’ task... I have to make an assumption that the additional condition ‘Piece LDF approved’ can actually be true: if it can be true this can be a valid trace. And I don’t know whether there was any kind of correlation here, there was no data object that was showing me this.*” Here, SP6 explained the approach taken to overcome the ambiguity by referring with *here* to multiple elements of the process model that were looked at while seeking for cues that could help to resolve the ambiguity. The analysis of the think-aloud data suggests that this behavior relates to an attempt to determine which are the useful elements, among a large set of elements, in the process model (e.g., an activity or a data object) able to provide contextual information to resolve the ambiguity. Looking at our entire dataset, *Explore* behavior, with similar characteristics, occurred in 41 trials.

***Target*** corresponds to long visits ($\ge 250ms$) to a rather limited number of specific process elements. For example, participant KP23 exhibited this behavior pattern when confronted with an ambiguous XOR gateway that could be interpreted either as a split or as a join (the ambiguous model fragment is depicted in Fig. 8, C). The AOIs-order over time plot, shown in Fig. 11, illustrates KP23’s behavior. In the plot, long visits to a few different process elements can be observed between the first and the last visit to the ambiguity. When asked about the approach taken to overcome the ambiguity, KP23 stated “*There was one outgoing decision and two incoming. Since I came from one of the paths, I just chose to use the one available decision to go to the end.*” Here, KP23 referred to using the information coming from the specific process elements that were looked at when confronted with ambiguity in order to resolve it. As it emerges from the analysis of the think-aloud data, this behavior suggests an attempt to integrate contextual information to resolve the ambiguity that is situated among a small set of clearly identified elements. Looking at our entire dataset, *Target* behavior, with similar characteristics, occurred in 82 trials.

***Bounce*** corresponds to back-and-forth transitions between the ambiguity and another specific process element. For example, participant SP20 exhibited this behavior pattern when confronted with ambiguity resulting from an abbreviation in an activity label (“M. p. TRS, XRG”), which could be interpreted in multiple ways (e.g., “Move pieces”, “Merge pieces”; the ambiguous model fragment is depicted in Fig. 8, B). The visual behavior of SP20 is visualized in the plot in Fig. 12. The plot shows alternating visits between the ambiguous element and a specific second process element, represented by turquoise bars in the figure. When asked about the approach taken to overcome the ambiguity, SP20 commented “*Because the box before says ‘Move piece’ [...] I just assumed that it means ‘Move piece’.*” Here, SP20 referred to using specifically the cue from the single process element (besides the ambiguity) that was looked at in back-and-forth transitions while trying to resolve the ambiguity. Informed by the think-aloud data analysis, this behavior suggests an attempt to leverage information from a single specific process element identified as useful to resolve the ambiguity. This could be, for example, an activity label hinting at a possible interpretation of an ambiguous abbreviation. Looking at our entire dataset, *Bounce* behavior, with similar characteristics, occurred in 9 trials.

***Hover*** corresponds to a (mostly) uninterrupted sequence of long visits ($\ge 250ms$) to the process elements affected by the ambiguity. For example, participant SP9 exhibited this behavior pattern when confronted with an ambiguity due to overlapping activities, which results in an unclear process continuation (the ambiguous model fragment is depicted in Fig. 8, E). SP9’s visual behavior is shown in the plot in Fig. 13. The plot shows a mostly uninterrupted sequence of multiple visits to the ambiguous process elements – the control flow edges in this case. When asked about the approach adopted to overcome the ambiguity, SP9 responded “*Everything seems to be kind of squished together and a bit messed up. And how do I solve it? I tried to decipher it anyways. So after ‘Inspect pieces BFV, LBG’ I could see that behind another one of those actions the arrow was still semi-transparently visible, and I think that the next step here is called ‘Bake piece HRT’*.” Here, SP9 explained the approach taken to resolve the ambiguity by stating that it involved closely inspecting the ambiguous elements of the process model, implying that this was the part of the model that involved major inspection. Following our analysis of the think-aloud data, this behavior suggests an attempt to understand and compare the possible interpretations of the ambiguous process fragment. Looking at our entire dataset, *Hover* behavior, with similar characteristics, occurred in 61 trials.

The examples shown in the AOIs-order over time plots in Figs. 9, 10, 11, 12 and 13 illustrate a dominating visual behavior pattern each. However, model readers confronted with ambiguity do not necessarily exhibit a single dominating visual behavior pattern. Indeed, in our analysis, we observed trials in which the participants exhibited multiple patterns, for example *Hover* interleaved with *Target* by SP3 when confronted with the ambiguity about overlapping activities, which resulted in *Hover* by SP9. The visual behavior of SP3 exhibiting multiple patterns is shown in the plot in Fig. 14. In the plot, two mostly uninterrupted sequences of long visits to the ambiguity are interleaved by long visits to few other process elements. When asked about the approach taken to overcome the ambiguity, SP3 stated “*It was not possible to see the options properly and it was very cluttered. It was very hard [...] I figured... I made an assumption on how the activities are connected.*”. Triangulating this comment with the observed visual behavior showed us that initially focusing on the ambiguity through a *Hover* behavior was not helpful for SP3, but then shifting to a *Target* behavior (focused on the connected activities) helped to figure some cues to make an assumption on the interpretation of the ambiguous model fragment that led to the completion of the task. The analysis of the entire dataset suggests that model readers do not always employ a single strategy, but apply multiple strategies while attempting to resolve ambiguity. Specifically, we observed *Sweep* behavior in combination with other behaviors in 79 trials, *Explore* behavior in combination with other behaviors in 60 trials, *Target* behavior in combination with other behaviors in 154 trials, *Bounce* behavior in combination with other behaviors in 42 trials, and *Hover* behavior in combination with other behaviors in 122 trials. Besides, in 4 out of our 342 trials, the participants’ behavior was unclear in the plots due to quality issues in the recorded data.

Moreover, for any given ambiguity, we did not consistently observe the same visual behavior pattern across the participants of our study. We also did not observe a consistent adoption of the same pattern across the ambiguities for any given participant.

## Discussion

### Testing the effects of ambiguity

Our findings with respect to RQ1 (cf. Introduction section), reveal that layout, semantic, and syntactic ambiguities, which affect the process control flow, exert a significant impact on cognitive aspects. These ambiguities increase model readers’ cognitive load, reduce their comprehension, and result in a significant amount of visual associations, indicating heightened cognitive integration effort (Bera et al. 2019). In contrast, lexical ambiguities, which affect the labels, exert a less pronounced effect. While we could observe effects in terms of lower comprehension and higher visual associations, no clear effects could be observed in terms of cognitive load. Specifically, the mean number of fixations with duration $\ge 250ms$, which are associated with mental processing (cf. Cognitive load, comprehension, and visual associations section), did not significantly differ between models with and without lexical ambiguities. A possible explanation of this fact is that ambiguities in the lexicon may not impose as much cognitive load as ambiguities in the control flow.

### Exploring ambiguity resolving behaviors

Our findings with respect to RQ2 (cf. Introduction section) suggest that readers exhibit different behaviors when resolving ambiguities. Specifically, we observed and defined for the first time five distinct patterns of visual behavior induced by ambiguity in process models, namely *Sweep*, *Explore*, *Target*, *Bounce*, and *Hover*. These behaviors suggest different strategies of attempting to integrate information to resolve ambiguities.

**Fig. 15:**
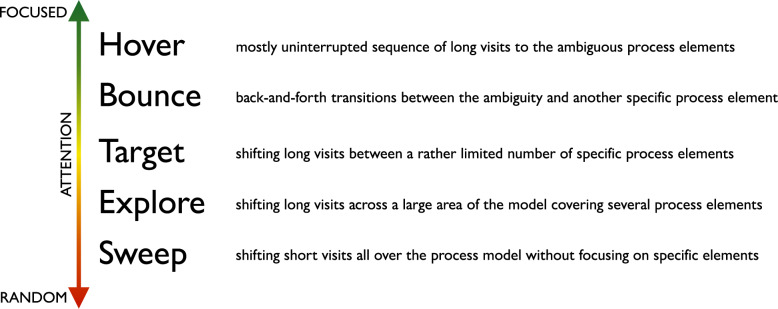
Visual behavior codes in a scale of attention. *Hover* suggests the most focused attention when facing ambiguity, while *Sweep* suggests the most random attention

Building upon the findings presented in Findings section and applying axial coding (Bryant and Charmaz 2019) (cf. Data collection and analysis section and the qualitative exploratory analysis process illustrated in Fig. 5), we examined the relationships between the identified visual behavior patterns and the levels of attention exhibited by the participants when resolving ambiguity. Herein, we suggest that the observed ambiguity-resolving behaviors are associated with varying degrees of attention focus. Figure 15 illustrates these degrees through a continuous scale of attention focus. In the scale, *Sweep* reflects the least focused and most random attention, involving unfocused visits spanning the whole process model. *Explore* denotes a dispersed attention, as it is characterized by a non-targeted exploration of multiple process model elements, suggesting that the readers’ attention is not focused toward specific process elements but toward general contextual information. *Target* is associated with an intermediate degree of attention, as model readers shift between a limited number of process elements, suggesting that the readers’ attention is focused toward identifying elements that can provide disambiguation cues from a selected pool of candidates. *Bounce* indicates a higher degree of focused attention, as model readers alternate between the ambiguous elements and a specific process element likely identified as relevant, suggesting that the readers’ attention is focused toward applying the information extracted from this element to resolve the ambiguity. Finally, *Hover* is associated with the highest level of focused attention, as model readers concentrate intensely on the ambiguous process elements while trying to resolve the ambiguity through a detailed element examination.

As reported in Findings section, we did not observe consistent associations between specific ambiguity types, visual behavior patterns, or participants. This suggests that the adoption of an ambiguity resolution strategy is rather subjective and varies between the concrete ambiguities a model reader is confronted with.

### Supporting model readers

Given the demonstrated negative effects of ambiguities, it becomes crucial to explore ways to better support model readers–either by minimizing ambiguities in process models or by enhancing the readers’ ability to handle them. For instance, automated techniques for label refactoring (cf. Pittke et al. (2015)) could help to lessen the presence of lexical ambiguity. However, it is unrealistic to assume that ambiguities can be completely eliminated. As a matter of fact, certain ambiguities are intentional, for example designed to enable flexible interpretation and execution of processes (cf. Franceschetti et al. (2023)). Despite this, the significant impact of ambiguities on task performance underscores the need for support beyond merely detecting modeling errors. One potential approach is fostering a feedback loop in which modelers and model readers collaborate to identify ambiguous process elements. Alternatively, process model analysis tools could be developed to automatically detect ambiguities. While not all ambiguities might be automatically detected due to their inherently subjective nature, one can envision tools for the automatic detection of certain syntactic ambiguities based on formal definitions, such as those presented in Ambiguity in process models section or in Franceschetti et al. (2023), or lexical ambiguities based on Natural Language Processing. These tools might leverage ontology annotations, similar to the techniques proposed in Fan et al. (2016).

Another possibility is that, with further development, the cognitive effects and behavior patterns associated with the detection and resolution of ambiguity could be leveraged to develop a new generation of context adaptive systems that deploy pre-trained machine learning models (e.g., Abbad-Andaloussi et al. (2024)) to detect when users are facing ambiguities and guide them in disambiguating the model, helping them, in turn, to move from random attention to focused one. This can, for example, be achieved by highlighting contextual information or displaying additional artifacts such as guiding annotations or simulations. Specifically, to detect ambiguities, the machine learning models can be trained using a supervised learning approach (Aggarwal 2015). Features derived from eye-tracking fixations, saccades, visits to AOIs (Holmqvist et al. 2011), and other cognitive and behavioral patterns associated with ambiguity can be collected within defined time windows and paired with labels indicating whether users are experiencing ambiguity. These labels may be obtained from think-aloud protocols conducted concurrently with the task. The models can be then trained to map the extracted features to the corresponding ambiguity label at the level of each time window. At run-time, incoming data can be buffered into time windows, each fed into the trained machine learning model to predict the presence of ambiguity. If an ambiguity is detected, then contextual support can be provided by highlighting relevant information or presenting additional artifacts, such as guiding annotations or interactive simulations.

### Implications

We identify implications for research, practice, and education underscored by our findings.

*Implications for Research.* Regarding research, future empirical studies examining human and cognitive aspects in process modeling should account for the presence of ambiguity in process models. Ambiguities could unintentionally be a confounding factor influencing research outcomes, making it essential to mitigate them in the design of future experiments. With the AOIs-order over time plots we introduced a visualization that emphasizes the visual behaviors adopted by model readers when confronted with ambiguity. This paper presents a first attempt at using these plots toward this goal, which resulted in the identification of key patterns describing how ambiguities are resolved. By capturing two essential aspects of process model comprehension, i.e., attention to different model parts and transitions between them, these plots offer a valuable tool for future studies. Specifically, they can support deeper exploration of users’ attention distribution (through the height of the bars referring to the time spent during each visit to a process model element) and cognitive integration processes (through the order of the bars referring to their order of occurrence over time), both critical for understanding process models (Bera et al. 2019).

*Implications for Practice.* Regarding practice, our results highlight the importance of minimizing ambiguities in process models as well as providing disambiguation cues to support model readers. This could be achieved by enriching process models with supplementary information (cf. Abbad-Andaloussi et al. (2021)) or making contextual cues in the models more explicit, since our qualitative study suggests that model readers tend to look for disambiguating cues in process models when they are confronted with ambiguity.

*Implications for Education.* With regards to education, our results underscore the importance of raising awareness among process modeling trainees about the potential ambiguities in their models and the impact these ambiguities can have. Training programs should incorporate discussions on identifying and managing ambiguities, empowering trainees with the skills to create process models that can easily be interpreted.

### Threats to validity

We recognize the existence of potential threats to the validity of our study. First, *internal validity* may be threatened by the presence of confounding factors that cannot be entirely eliminated. However, we mitigated this threat by designing a controlled experiment based on a pre-defined research model (cf. Study design section), carefully and systematically preparing the experiment materials (cf. Study design section), randomizing the order in which the tasks were presented to avoid learning and fatigue effects (cf. Experiment procedure section), and adhering to a strict and uniformly applied data collection protocol during all sessions of data collection (cf. Experiment procedure section).

A second threat to internal validity arises from retrospectively asking participants to state whether they encountered any issues while performing each task after its completion. Each task was based on a process model that was deliberately designed to include an ambiguity that hindered its execution. Therefore, it is possible that the participants might have anticipated the presence of issues in subsequent tasks due to this repeated questioning. Such anticipation represents a potential confounding factor, as it could influence their cognitive processing. To mitigate this threat, we took several measures. We carefully avoided disclosing the goals of our study to the participants, and avoided mentioning that they would encounter issues while analyzing the process models. We refrained from making the participants aware of the concept of ambiguity in process models, never mentioning ambiguity. Additionally, we deliberately phrased the question in a generic manner to avoid suggesting that participants were being asked to analyze process models affected by ambiguity (*“Name any issues you encountered while answering the question, along with how you overcame them”*). Finally, by randomizing the presentation of the tasks (cf. Study design section) we ensured that the increased cognitive load resulting from the potential anticipation of issues was evenly distributed across all tasks. Nevertheless, in future studies, this threat could be further mitigated with the inclusion of tasks where all the models are free from ambiguity and by phrasing questions excluding nudging terms such as *issues* (e.g., *“Name any difficulties you encountered while answering the question, along with how you overcame them”*), thereby minimizing the likelihood of participants anticipating and actively seeking out problems.

With regards to our qualitative analysis, a potential threat to internal validity derives from the possibility that the visual behaviors do not accurately capture the approaches employed by the participants to resolve ambiguity. To mitigate this threat, we triangulated the visual behavior patterns with the retrospective think-aloud data of the study participants when asked about the strategies adopted to overcome the difficulties in solving the given tasks. Another threat stems from potential bias in our subjective interpretation of the visual behavior patterns, represented through the AOIs-order over time plots, and the insights, extracted from the think-aloud data. To mitigate this threat, we reviewed the coding in order to validate our interpretations (cf. Data collection and analysis section). Moreover, we recognize the limitation of measuring only one of the two components of comprehension efficiency, namely the response time, neglecting comprehension accuracy. As explained in Cognitive load, comprehension, and visual associations section, this is due to the impossibility to measure comprehension accuracy due to the inherent admissibility of the multiple interpretations of ambiguities.

The *external validity* of our study may be threatened by the inability to generalize the experiment results due to the sample size of the participants or the modeling language used in the process models. To mitigate these threats, we recruited 44 participants, which, to the best of our knowledge, positions our study among the most extensive eye-tracking studies conducted in the context of process modeling. Regarding the specific use of BPMN, we argue that the investigated ambiguities are not exclusive to BPMN. Such ambiguities could also be encountered in other imperative modeling languages, such as workflow nets and EPC models (Keller et al. 1992).

## Conclusion

Ambiguities in a process model result in multiple potential interpretations of the process. By leveraging eye-tracking, we explored how these ambiguities influence model readers during various model comprehension tasks. Our findings reveal a significant influence on cognitive load, comprehension, and visual associations. Additionally, our results indicate the adoption of specific visual behavior patterns when solving ambiguity (*Sweep, Explore, Target, Bounce,* and *Hover*), which can be associated with different levels of attention ranging from random to focused attention.

The negative effects of ambiguity highlight the importance of providing adequate training and exercising caution to minimize ambiguities in process models. Additionally, they underscore the need for designing novel automated tools to assist model readers in identifying ambiguities and potentially resolving them. In future work, it is worthwhile to investigate the role played by BPM expertise in the detection of ambiguities, including in relation to diverse industrial backgrounds. Furthermore, it is also worthwhile to specifically investigate the cognitive and behavioral impact of intentional ambiguities. Moreover, given the richness of our eye-tracking data, new machine learning models can be developed to automatically detect when readers are challenged with ambiguity, and guide them resolving it. These models will provide the foundation for a new generation of ambiguity-aware context-adaptive systems.

## Data Availability

An online appendix containing supplementary material as detailed in Data availability and reproducibility section is available at https://doi.org/10.5281/zenodo.16738478.

## Footnotes

1 In our previous work (Franceschetti et al. 2024), this ambiguity is called *pragmatic*. Here, we use the more specific term *layout* to better mark the distinction from pragmatic aspects in linguistics, which are not concerned with layout.

2 See https://camunda.com/download/modeler/

3 A trial refers to an instance of a participant performing a task
